# P11 promoter methylation predicts the antidepressant effect of electroconvulsive therapy

**DOI:** 10.1038/s41398-017-0077-3

**Published:** 2018-01-22

**Authors:** Alexandra Neyazi, Wiebke Theilmann, Claudia Brandt, Tomi Rantamäki, Nobuaki Matsui, Mathias Rhein, Johannes Kornhuber, Malek Bajbouj, Wolfgang Sperling, Stefan Bleich, Helge Frieling, Wolfgang Löscher

**Affiliations:** 1 Department of Psychiatry, Social Psychiatry and Psychotherapy, Medical School Hannover, Hannover, Germany; 2 Department of Pharmacology, Toxicology, and Pharmacy, University of Veterinary Medicine Hannover, Hannover, Germany; 3 Center for Systems Neuroscience, Hannover, Germany; 4 Division of Physiology and Neuroscience, Department of Biosciences, University of Helsinki, Helsinki, Finland; 5 Neuroscience Center, University of Helsinki, Helsinki, Finland; 6 Faculty of Pharmaceutical Sciences, Tokushima Bunri University, Tokushima, Japan; 7 Department of Psychiatry and Psychotherapy, Friedrich-Alexander-University of Erlangen-Nuremberg, Erlangen, Germany; 8 Department of Psychiatry, Charité Universitätsmedizin Berlin, Campus Benjamin Franklin, Berlin, Germany

## Abstract

Although electroconvulsive therapy (ECT) is among the most effective treatment options for pharmacoresistant major depressive disorder (MDD), some patients still remain refractory to standard ECT practise. Thus, there is a need for markers reliably predicting ECT non/response. In our study, we have taken a novel translational approach for discovering potential biomarkers for the prediction of ECT response. Our hypothesis was that the promoter methylation of p11, a multifunctional protein involved in both depressive-like states and antidepressant treatment responses, is differently regulated in ECT responders vs. nonresponders and thus be a putative biomarker of ECT response. The chronic mild stress model of MDD was adapted with the aim to obtain rats that are resistant to conventional antidepressant drugs (citalopram). Subsequently, electroconvulsive stimulation (ECS) was used to select responders and nonresponders, and compare p11 expression and promoter methylation. In the rat experiments we found that the gene promoter methylation and expression of p11 significantly correlate with the antidepressant effect of ECS. Next, we investigated the predictive properties of p11 promoter methylation in two clinical cohorts of patients with pharmacoresistant MDD. In a proof-of-concept clinical trial in 11 patients with refractory MDD, higher p11 promoter methylation was found in responders to ECT. This finding was replicated in an independent sample of 65 patients with pharmacoresistant MDD. This translational study successfully validated the first biomarker reliably predicting the responsiveness to ECT. Prescreening of this biomarker could help to identify patients eligible for first-line ECT treatment and also help to develop novel antidepressant treatment procedures for depressed patients resistant to all currently approved antidepressant treatments.

## Introduction

Depression is a major cause of disability worldwide; however, despite several decades of intense research, our understanding of the pathophysiology of major depressive disorder (MDD) remains limited^1,2^. Treatment with antidepressant drugs is ineffective in up to two-third of depressed patients and, when effective, delayed in onset and afflicted with side effects^3,4^. Thus, there is a major unmet medical need for more effective therapies. Electroconvulsive therapy (ECT) remains the most effective treatment option for drug-refractory depressed patients, but its mechanisms still remain elusive^5–7^. Furthermore, approximately one third of pharmacoresistant depressive patients are resistant to ECT as well^8,9^. Biomarkers reliably predicting the response to ECT would not only significantly reduce costs and identify patients eligible for first-line ECT treatment but also help to develop novel ECT and other treatment procedures for a subgroup of depressed patients resistant to currently approved antidepressant treatments.

The overall aim of the present study was to identify potential biomarkers for ECT response by developing a valid rat model of pharmacoresistant depression and then translate the experimental findings to depressive patients in a proof-of-concept (POC) clinical study. Validated animal models of depression are based on the observation that chronic stress is a central causal factor for the development of MDD^10^. The chronic mild stress (CMS) model was developed to mimic anhedonia—one of the core symptoms of MDD^11^. We adapted this model with the aim to obtain rats that are resistant to conventional antidepressant drugs (citalopram). Afterwards, such animals were treated with two different types of ECT as recently described^12^. Based on a recent study with this animal model of depression^13^, showing a rat breeder-dependent effect of CMS on p11 methylation, our hypothesis was that the expression and promoter methylation of p11, a member of the S100 EF-hand family^14^, might be differently regulated in ECT responders compared with nonresponders and thus be a putative biomarker of ECT response. This hypothesis is supported by several lines of evidence. In our recent rat study, vulnerability and resilience of different Wistar rat substrains to CMS were reflected in epigenetic regulation and expression of p11^13^. Moreover, p11 is decreased in prefrontal cortex (PFC), hippocampus, and nucleus accumbens of depressed patients and in animal models of depression, whereas antidepressants and ECT increase p11 in the rodent brain^15^. P11 amplifies serotonin receptor-mediated signaling and regulates gene transcription^16^. The precise mechanisms underlying the antidepressant therapy-associated increase in p11 levels remains obscure, but they appear to involve an upregulation of brain-derived neurotrophic factor (BDNF) signaling^15^. Indeed, BDNF and its epigenetic regulation have been associated with both the pathophysiology of depression and the mode of action of antidepressant treatments^17–21^. In line with this concept, we recently reported that pharmacoresistant depressive patients remitting under ECT had significantly lower mean promoter methylation rates of the BDNF gene compared with nonremitters^22^. Therefore, we also included BDNF promoter methylation measurements in the present animal experiments.

## Materials and methods

See Supplementary Material for additional details.

### Preclinical experiments

**Fig. 1:**
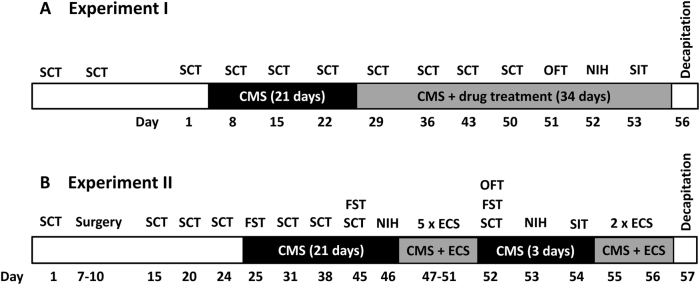
Schematic protocol of the two preclinical experiments. In experiment I **a**, the effects of citalopram on CMS-induced behavioral alterations were determined, whereas the effects of ECS were evaluated in experiment II **b**. Abbreviations: CMS, chronic mild stress; ECS, electroconvulsive stimulation; FST, forced swim test; NIH, novelty-induced hypophagia; OFT, open field test; SCT, sucrose consumption test; SIT, social interaction test

The design of the preclinical experiments is illustrated in Fig. 1. For the preclinical experiments, 46 male Wistar outbred rats (Charles River, Sulzfeld, Germany) underwent the CMS protocol, 10 age-matched male Wistar rats served as unstressed controls. We have previously shown^13^ that Wistar outbred rats from this vendor exhibit increased anxiety-related behavior and increased response to CMS compared with such rats from other vendors so that we expected that CMS-induced behavioral alterations induced in this Wistar substrain (Crl : WI(Han)) might be particularly severe and difficult to treat by antidepressants. Depression-like symptoms were measured by a battery of behavioral tests, as one behavioral test alone might not capture a more complete picture of the effects of CMS, citalopram, and the ECT treatment. This battery included the sucrose consumption test (SCT), the forced swim test, novelty-induced hypophagia, the open-field test, and the social interaction test. As in the experiments of Christensen et al.^23^, sucrose consumption rather than sucrose preference was measured, because the SCT has been established by Wiborg and colleagues^23^ as a well-suited measure to select for responders and nonresponders to antidepressive treatment. All rats were randomly assigned to treatment groups. Electroconvulsive stimulation (ECS) was applied as previously described^12^. Classification of treatment response was defined by the within-subject change in sucrose consumption according to Christensen et al.^23^. Rats were anesthetized and decapitated 24 h after the last (sham)-ECS, citalopram, or vehicle treatment for analysis of p11 and BDNF in the PFC. See Supplementary Material for details of the animal experiments, biochemical analyses, and statistical evaluation of data.

### Clinical experiments

A prospective POC clinical study was performed in 11 in-patients with a pharmacoresistant depression receiving ECT, recruited at the University Hospital of Erlangen (Ethics number: 3252/2006). Response was defined by a ≥ 50% reduction of the Montgomery Asberg Depression Scale. Fasting blood samples were taken directly before (8–10 a.m.) ECT sessions 1, 4, 7, and 10. For the independent replication sample, 67 in-patients with a pharmacoresistant depression receiving ECT were acquired at the Department of Psychiatry of the Charité Berlin, to assure the blinded performance of the molecular analyses (Ethics number: EK-224-05c). Depression severity was assessed before and weekly (for 6 weeks) during ECT treatment. A ≥ 50% reduction of the Hamilton Depression Rating Scale (HAMD) within the treatment period was interpreted as response to therapy: Fasting blood samples (n = 65) were taken once before the initation of the ECT treatment series. There were no outliers excluded from the analyses. Antidepressant and antipsychotic medication (as well as total leucocyte counts measured in the replication sample) did not differ between responders and non-responders to therapy. In both clinical studies, informed consent was obtained after the nature and possible consequences of the studies were explained. See Supplementary Material and Supplemental Tables S1 and S2 for details of the clinical trials, biochemical analyses, and statistical evaluation of data.

## Results

### CMS-induced alterations in sucrose consumption in rats

**Fig. 2:**
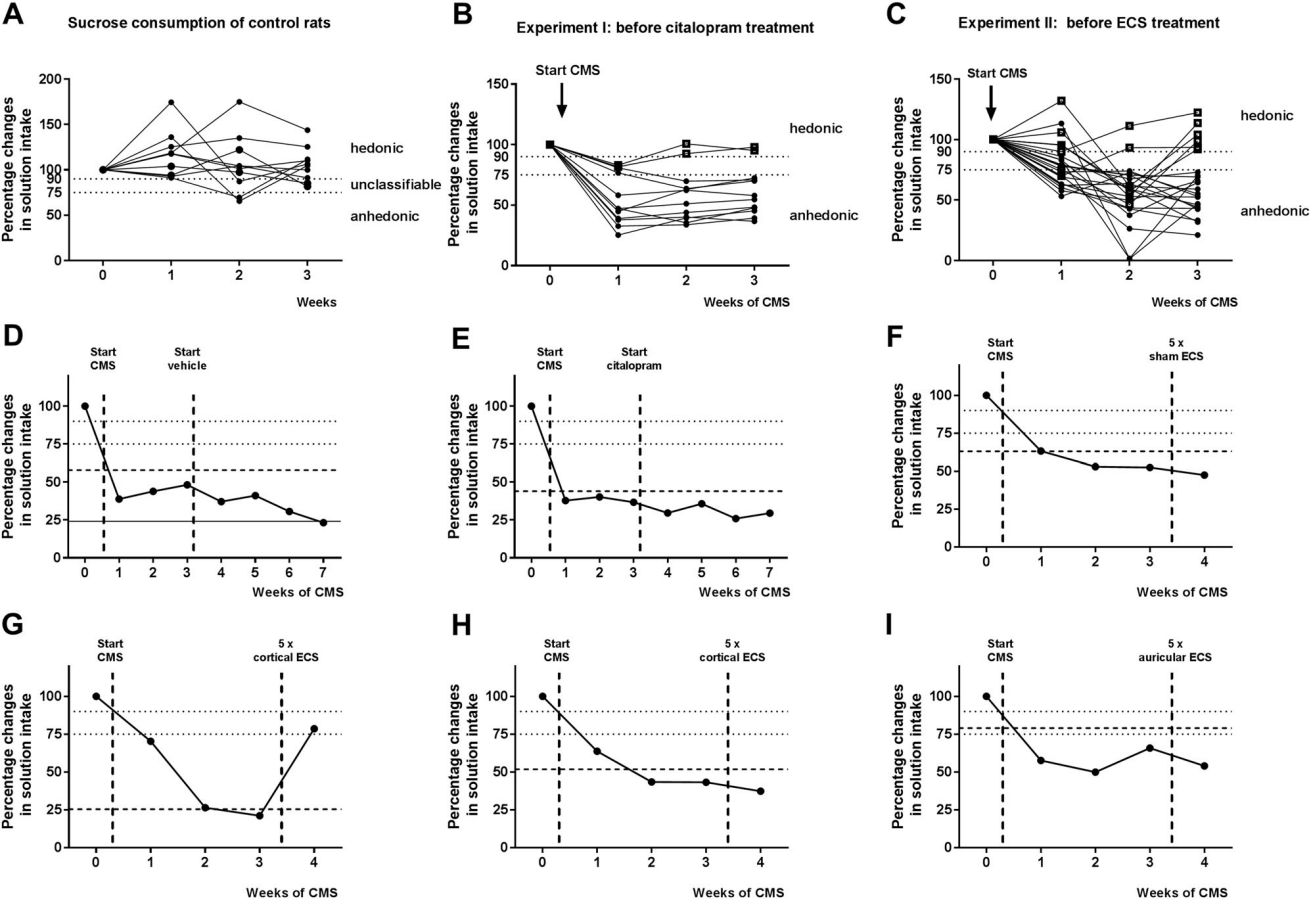
Individual responses to chronic mild stress (CMS) and antidepressive treatments in the sucrose consumption test in rats. **a** Illustrates that sucrose consumption survey over 3 weeks in unstressed control rats results in 8 out of 10 rats with hedonic-like behavior, whereas 2 rats were unclassifiable. **b** Data from experiment I, showing that 3 weeks of CMS induced anhedonic-like behavior in 10 out of 15 animals. Thus, the number of anhedonic-like rats was increased after CMS compared with unstressed controls (*P* = 0.0024). Hedonic-like behavior was present in 2 out of 15 animals. **c** Data of experiment II, showing that 3 weeks of CMS-induced anhedonic-like behavior in 17 out of 29 rats. Thus, the number of anhedonic-like rats was increased after CMS compared with unstressed controls (*P* = 0.0015). Hedonic-like behavior was present in 6 out of 29 rats. Small dashed lines represent threshold for selection of hedonic- and anhedonic-like rats (anhedonic-like rats > 25% within-subject decrease in sucrose consumption, hedonic-like rats < 10% within-subject decrease in sucrose consumption). **d**–**i** Representative data from individual rats following different types of antidepressive treatment. **d** A vehicle-treated rat from experiment I; **e** a citalopram-treated non-responder from experiment I; **f** a rat from experiment II after five sham ECS sessions; **g** a responder from experiment II after five cortical ECS sessions; **h** a nonresponder from experiment II after five cortical ECS sessions; and **i** a nonresponder from experiment II after five auricular ECS sessions. Responses of all treated rats are shown in Table 1. Wide dashed lines represent the threshold for positive response and solid lines represent threshold for negative treatment response. See Figs. S1–S5 for data of all individual rats

The 3-week sucrose consumption course of unstressed control rats demonstrated that none of the control animals showed anhedonic-like behavior (Fig. 2a and Table 1). Stressed rats were classified after 3 weeks of CMS as anhedonic-like or hedonic-like, based on changes in intake of sucrose solution. According to Christensen et al.^23^, anhedonic-like rats are supposed to show a > 25% within-subject decrease in sucrose consumption. Hedonic-like rats are supposed to show a < 10% within-subject decrease in sucrose consumption. Animals not responding to either criterion are considered as unclassifiable. In experiment I, CMS-induced anhedonic-like behavior was present in 66.7% (10/15) of the animals, whereas hedonic-like behavior was detected in 13.3% (2/15) of the rats (Fig. 2b and Table 1). Equally, about 60% (17/29) of CMS-exposed rats from experiment II showed anhedonic-like behavior and 21% (6/29) of the rats were classified as hedonic-like after CMS (Fig. 2c and Table 1). The average weekly body weight gain during three weeks of CMS was 7.6 g (range 3.3–10.6 g), so that weight gain of all CMS-exposed rats was below the average weight gain of naïve male Wistar rats of 15 g per week.

**Table 1:** Results from sucrose consumption test of the two experiments illustrated in Figs 1 and 2

Response in sucrose consumption test	Experiment I				Experiment II			
	Controls without CMS (*n* = 10) Exp. #1a	CMS			CMS			
		Citalopram			ECS			
		Before treatment (*n* = 15) Exp. #1b	After treatment		Before treatment (*n* = 29) Exp. #2a	After treatment		
			Vehicle (*n* = 8) Exp. #1c	Citalopram (*n* = 7) Exp. #1d		Sham (*n* = 9) Exp. #2b	Cortical ECS (*n* = 10) Exp. #2c	Auricular ECS (*n* = 10) Exp. #2d
Hedonic	8	2	1	0	6	3	6	2
Anhedonic	0	10	7	7	17	6	2	8
Unclassifiable	2	3	0	0	6	0	2	0
Significance of differences in proportion of hedonic rats (Fisher’s exact test)		*P* = 0.0024 vs. #1a	NS vs. #1b	NS vs. #1b	*P* = 0.0015 vs. #1a	NS vs. #2a	*P* = 0.0427 vs. #2a	NS vs. #2a

As described in Methods, the animals were randomly divided into the treatment groups and it was ensured that the number of anhedonic-like, hedonic-like, and unclassifiable rats were almost the same in all treatment groups

### Effects of citalopram on sucrose consumption in the CMS model in rats (experiment I)

Chronic citalopram treatment did not increase the intake of sucrose solution in any of the preassigned anhedonic-like rats (Table 1) so that they were classified as sub-categorical nonresponders. Examples of vehicle and citalopram-treated anhedonic-like rats are shown in Fig. 2d, e; data from all individual rats are shown in Figs S1 and S2. No behavioral adverse effects were observed during treatment with citalopram, but rats lost body weight compared to vehicle controls. One hedonic-like and one unclassifiable animal became anhedonic-like during citalopram (Fig. S2).

### Effects of ECS on sucrose consumption in the CMS model in rats (experiment II)

As shown in Fig. 1b, ECS was delivered once daily for 5 days. Afterwards, behavioral investigations were conducted before ECS was applied for further 2 days. CMS was continued during the stimulation period. After five ECS treatments, rats were classified according to their changes in sucrose intake (Table 1). Examples of sham and ECS-treated anhedonic-like rats are shown in Fig. 2f–i; data from all individual rats are shown in Figs S3–S5. Sham ECS exerted no significant effects in anhedonic-like rats (Fig. 2f). After cortical ECS, 67% (four of six) of anhedonic-like rats showed positive response (Fig. 2g and Table 1), whereas two of these rats did not respond (Fig. 2h). In contrast, about 80% (5/6) of anhedonic-like animals, which received auricular ECS, were characterized as nonresponders. Overall, cortical ECS was the only treatment that exerted a significant antidepressive effect in this rat model of drug-resistant chronic depression (Table 1).

As previously described in Theilmann et al.^12^, cortical and auricular ECS induced mainly generalized convulsive seizures of at least 15 s duration (mean seizure duration ( ± SEM) cortical ECS: 25.5 ( ± 1.35) s, mean seizure duration ( ± SEM) auricular ECS: 21.9 s ( ± 0.97)), as evident in the cortical electroencephalogram (EEG). Rats with cortical ECS showed predominantly generalized clonic seizures, whereas auricular ECS induced mainly tonic extensions of fore- and hindlimbs. We could also replicate our previous finding^12^ that significantly (*P* = 0.025) more rats with auricular ECS (7/10 animals) emitted calls of fear and distress (22 kHz) before and after stimulation than cortically stimulated rats (2/10 animals).

### Effects of ECS on other depression-like symptoms in the CMS model in rats

**Fig. 3:**
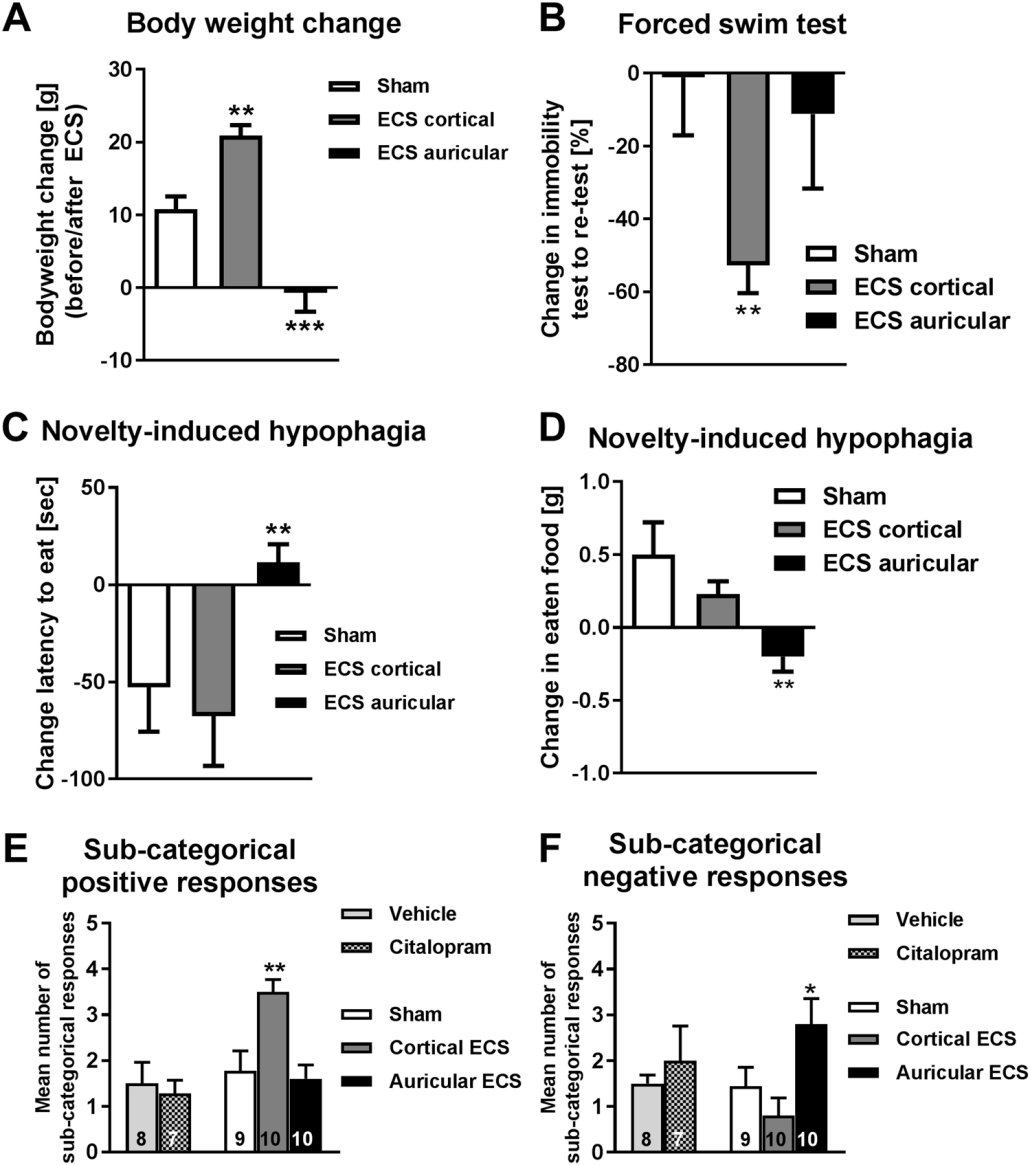
Effect of ECS in behavioral tests other than sucrose consumption and overall responses to antidepressive treatments in rats. **a**–**d** Grouped behavioral assessment after sham ECS, cortical ECS, and auricular ECS arranged by treatment groups. Data are shown as means ± SEM of 9 (sham), 10 (cortical ECS, and 10 (auricular ECS) rats. **a** Weight changes (g) (**P < 0.01, ***P < 0.001, comparing sham with auricular and cortical ECS) before and after ECS sessions. **b** Percentage change of immobility time in the forced swim test from test to re-test session. Test session was performed 2 days before ECS started. Re-test trial was performed 1 day after the fifth ECS (**P < 0.01, comparing sham with auricular and cortical ECS). **c** Change in latency to eat (s) (**P < 0.01, comparing sham with auricular and cortical ECS) from novelty-induced hypophagia test to re-test session. **d** Food intake (g) (**P < 0.01, comparing sham with auricular and cortical ECS) from novelty-induced hypophagia test to re-test session. Test session in **b** and **c** was performed 1 day before ECS. Re-test trial was performed 2 days after the fifth ECS. **e** and **f** Summary of treatment effects caused by vehicle vs. citalopram treatment and sham, cortical, and auricular ECS, including data from all tests shown in Table S3. **e** Total number of direct treatment responses. Vehicle and citalopram groups did not differ in responses (*P* = 0.903). Cortical ECS induced significantly more treatment responses compared with sham ECS (**P < 0.01), whereas auricular ECS was ineffective. **f** Total number of direct negative treatment responses. There were no significant differences between rats with vehicle and citalopram (*P* = 0.952). Auricular ECS was associated with significantly more negative responses than sham or cortical ECS (*P = 0.05)

In addition to the positive effect on sucrose consumption in the CMS model, cortical ECS significantly increased body weight, whereas it was decreased by auricular ECS (Fig. 3a). Furthermore, cortical, but not auricular ECS, decreased the duration of immobility in the forced swim test (Fig. 3b), a classical test assessing antidepressant-like behavioral responses. Auricular ECS exerted negative effects on novelty-induced hypophagia (Fig. 3c, d), adding to the overall negative response to this type of ECS. No significant intergroup differences were seen in the open field (time in center; *P* = 0.317) and social interaction tests (distance between animals, *P* = 0.981; time in body contact, *P* = 0.613) (not illustrated). When sub-categorical positive responses were calculated from all behavioral tests, cortical ECS was the only effective antidepressant treatment in the CMS model (Fig. 3e), whereas auricular ECS induced significantly more negative responses than sham treatment (Fig. 3f), and citalopram was not effective in any of the tests (Fig. 3e, f).

It is interesting to note that sham-treated animals, as a group, appeared anhedonic in the SCT after CMS (Fig. 2f; Table 1) but less anxious/depression-like in the novelty-induced hypophagia (Fig. 3c). Given that depression is a heterogeneous and complex disorder, it is unlikely to be that all CMS-exposed animals with hedonic disturbance show necessarily depressive-like behaviors in other depression-related symptoms and vice versa. Although the SCT and the novelty-induced hypophagia test are both based on the consumption of palatable nutrients, they still differ in other behavioral components, which might have been the crucial components for the rats to perform differently in one test compared with the other.

### Correlation between antidepressive response of ECS in the CMS model and alterations in p11 or BDNF

**Fig. 4:**
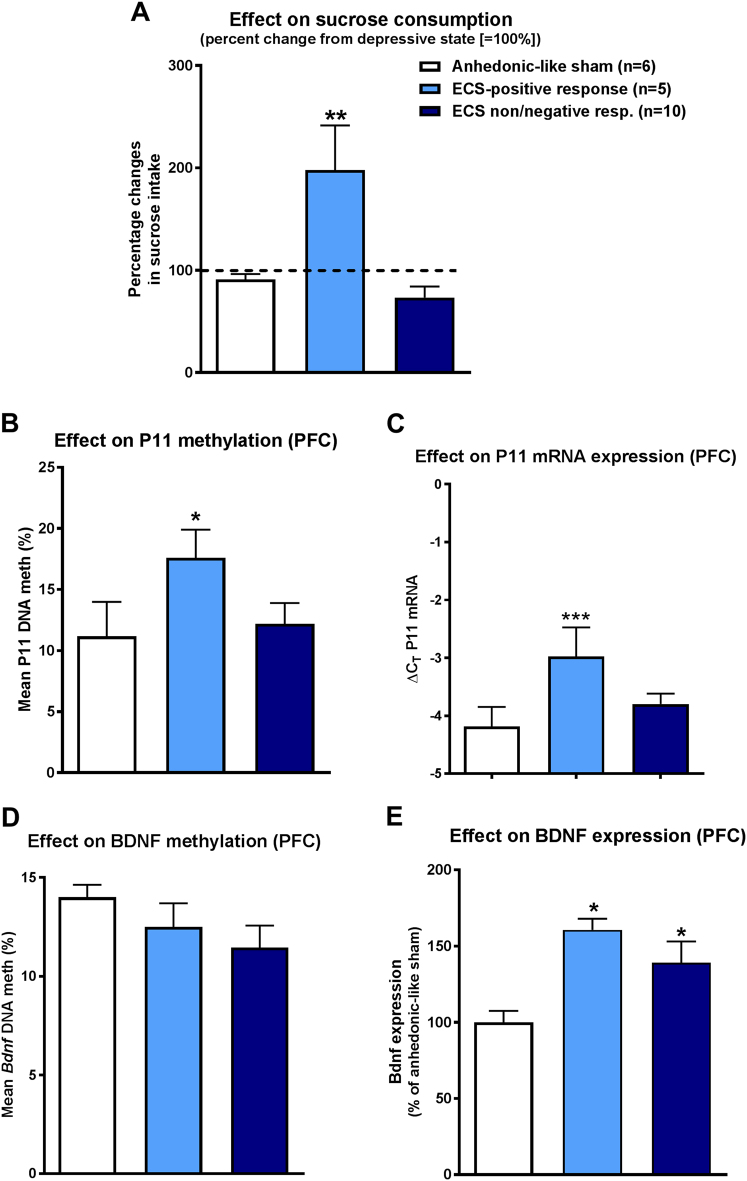
Association between ECS responses in the sucrose consumption test (SCT) in rats and p11 and BDNF expression and promoter methylation in the prefrontal cortex (PFC). As shown in **a**, three groups of rats were compared: anhedonic-like sham controls, rats with ECS-positive response in the SCT, and rats with no or negative response to ECS in the SCT. Data are shown as means ± SEM of 6 (anhedonic-like sham), 5 (positive ECS response), and 10 (no or negative ECS response) rats. In **a**, the percent change in sucrose intake following ECS treatment in responders was significantly different from sham and non/negative responders (**P < 0.01, comparing anhedonic-like sham animals with positive responders and non/negative responders). **b** Significant difference of the three groups regarding p11 methylation in PFC (* *P* = 0.017). **c** Similarly, such an effect was seen for p11 mRNA expression in PFC (***P < 0.001 comparing positive responders with anhedonic-like sham animals and *P* = 0.004 comparing positive responders and non/negative responders). **d** BDNF methylation was not different between groups (*P* = 0.284, comparing anhedonic-like sham animals with positive responders and non/negative responders), whereas ECS responders and non/negative responders **e** significantly differed from anhedonic-like sham rats in BDNF expression in PFC (*P < 0.05)

In order to study whether alterations in p11 or BDNF were associated with the antidepressive effect of ECS in the CMS rat model, we compared three groups of rats: anhedonic-like sham controls, rats with ECS-positive response in the SCT, and rats with no or negative response to ECS in the SCT (Fig. 4). As shown in Fig. 4a, the percent change in sucrose intake following ECS treatment in responders was significantly different from sham and non/negative responders. P11 promoter methylation was significantly increased in the PFC of ECS responders (Fig. 4b). Furthermore, p11 mRNA expression was significantly higher in PFC of responders (Fig. 4c). In contrast to p11, BDNF did not discriminate between ECS responders and nonresponders (Fig. 4d, e). As shown in Fig. 4d, BDNF promoter methylation did not differ among groups, while both ECS groups exhibited significantly higher BDNF protein expression than sham controls (Fig. 4e).

### POC study in patients with pharmacoresistant MDD

Based on the preclinical data, we examined whether patients (*N* = 11) differing in their response to ECT also differ in peripheral p11 promoter methylation. Patients’ baseline characteristics are shown in Table S1. At the end of the ECT series four patients had responded to ECT.

**Fig. 5:**
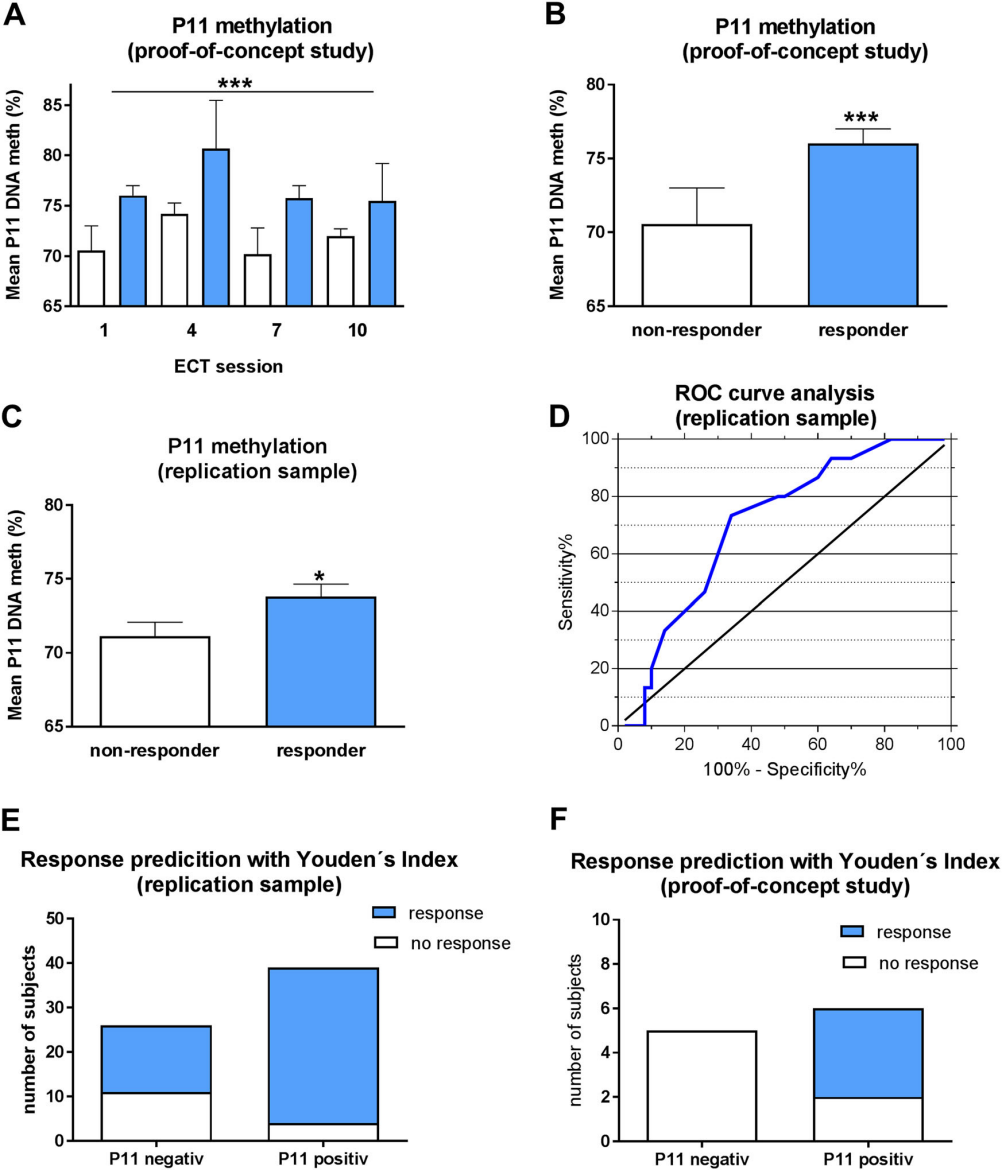
Association between clinical response to ECT and p11 promoter methylation. Data are shown as means ± SEM; significant differences between groups are indicated by asterisks (*P < 0.05; **P < 0.01; ***P < 0.0001). As shown in **a** and **b**, p11 promoter methylation was higher in responders (*n* = 4) compared with nonresponders (*n* = 6) directly before ECT sessions 1, 4, 7, and 10 (response: *P* < 0.0001; ECT-number: *P* = 0.094, derived from mixed linear modelling including the factors age and gender). **c** Higher baseline p11 promoter methylation of responders (*n* = 50) compared with nonresponders (*n* = 15) to ECT in the independent clinical replication sample (*P* = 0.014, derived from mixed linear modelling including the factors age and gender). **d** ROC curve analysis to evaluate the efficiency of p11 promoter methylation levels in differentiating responders fron nonresponders in the independent clinical replication sample with area under the curve values (AUC) of 0.711 (95% CI: 0.575–0.846; *P* = 0.006). Values of above the threshold of 72.15% (derived from Youden’s Index) were robust to discriminate responders from nonresponders with a sensitivity of 70% and specifity of 73% as shown in **e**. P11 hypermethylation predicted ECT response with a probability of 89%. **f** The derived cutoff from Youden’s Index was sufficient to distinguish responders (all above the threshold of 72.15%) from nonresponders in the POC study (*P* = 0.045)

Mixed linear modelling showed no change in p11 promoter methylation over time but revealed differences between responders and nonresponders: p11 promoter methylation was significantly higher in responders than in nonresponders before each ECT (Fig. 5a, b; for methylation at different CpG sites see Supplemental Figure S10A).

### Prediction of response in an independent clinical replication sample

Based on the POC study, p11 promoter methylation was studied in an independent replication sample of 65 pharmacoresistant patients receiving ECT. Baseline characteristics of the replication sample are shown in Table S2. We could replicate our finding of higher baseline p11 promoter methylation in responders to ECT compared with nonresponders (Fig. 5c; for methylation at different CpG sites see Supplemental Figure 10B).

**Fig. 6:**
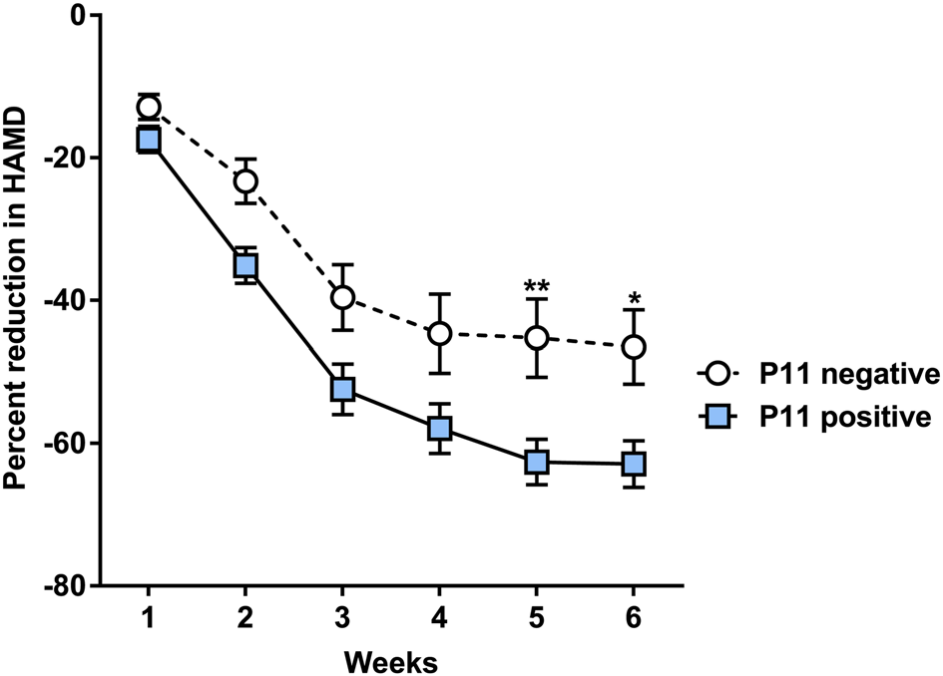
HAMD score reduction and baseline p11 methylation status. Significant differences between groups are indicated by asterisks (*P < 0.05; **P < 0.01; ***P < 0.0001). P11-positive patients at baseline (methylation above Youden’s Index cutoff, *n* = 39) showed a significantly higher reduction in the HAMD score when compared with p11-negative patients (*n* = 26) at week 5 and 6

Next, we performed receiver operating characteristic (ROC) analyses to validate the potential usefulness of p11 promoter methylation to predict response to ECT. Our ROC curves revealed that p11 promoter methylation levels above the threshold of 72.15% were robust to discriminate responders from nonresponders with AUC values of 0.711 (95% confidence interval (CI): 0.575–0.846; Fisher’s exact test *P = *0.006, Fig. 5d). The sensitivity and specificity to identify responders was 70% and 73%, respectively (Fig. 5e). The odds ratio for patients with serum p11 promoter methylation cutoff threshold values of > 72.15% to respond to ECT was 6.417 (95% CI: 1.759–23.413). P11 hypermethylation predicted ECT response with a probability of 89%. Furthermore, there was a significant difference in the percentage of HAMD score reduction between p11-positive and p11-negative patients at week 5 (Bonferroni corrected post-hoc tests following two-way analysis of variance *P* < 0.05) and 6 (*P* < 0.01) (Fig. 6).

To further confirm the reproducibility of p11 promoter methylation as a marker for response to ECT we applied the derived cutoff from Youden’s Index to our POC study and could distinguish responders from nonresponders to ECT (Fisher’s exact test (one-sided) *P = *0.045, Fig. 5f). All responders had p11 methylation levels above the threshold of 72.15%.

## Discussion

The CMS model of depression^24^ is a highly validated method to reflect symptoms (endophenotypes) of human MDD in rats and to analyze antidepressant therapy effects. As a core symptom of MDD, CMS causes changes in hedonic-like behavior, which are mirrored by decreased intake or preference of sweetened solution. Some studies demonstrated CMS-induced segregation of rats in subgroups that either show anhedonic-like behavior or revealed resilience to CMS^23,25,26^, which was also observed in the present study. Moreover, CMS induces various other depression-like symptoms, including increased behavioral despair (e.g., immobility in the forced swim test) and learned helplessness, decreases in self-care, loss of body weight, and sleep changes^27^. Almost every study that has examined the effects of chronic treatment with antidepressant drugs, including citalopram or escitalopram, has reported that such drugs were effective in reversing or preventing these ‘depressive’ behavioural changes in the CMS model^27^, although some studies reported that a subgroup of the treated rats was resistant to antidepressant drugs^23,26,28^. By using a substrain (Crl:WI(Han)) of male outbred Wistar rats that is particularly stress-sensitive^13^, we established a CMS model in which all examined rats were resistant to citalopram, thus establishing a novel rat model of drug-resistant chronic depression that can be used to explore the efficacy of alternative treatments such as ECS and to discover biomarkers predicting ECS response. Our recent study in outbred Wistar substrains from different vendors had shown that the differences to CMS in these substrains are reflected in epigenetic regulation and expression of p11^13^, so that p11 seemed to be a good candidate for the aims of the present ECS study.

Treatment-resistant depression or treatment-refractory depression is a term used in clinical psychiatry to describe cases of MDD that do not respond adequately to appropriate courses of at least two antidepressants^3,4^. Thus, the fact that we included only one drug, citalopram, in the present rat experiments is a limitation of the model and we plan to characterize the drug resistance of the model in more detail in the future. Furthermore, an independent replication of the preclinical findings with this complex chronic model would be helpful, as this experiment has been done only once. Studies on the effect of ECS (the analog to ECT in animals) in validated animal models of depression are sparse; however, repeated auricular ECS treatments have been shown to reverse stress-induced anhedonia in the CMS rat model^29,30^. In the present study, cortically applied ECS was clearly more effective than citalopram to reduce anhedonia, which is in line with clinical experience. However, as already noted by Henningsen et al.^30^, not all rats responded to ECS so that responders and nonresponders could be selected. When comparing cortical ECS with auricular ECS, as used in the studies of Moreau et al.^29^ and Henningsen et al.^30^ and most other ECS studies in animals, cortical ECS was clearly more effective to reduce depression-like symptoms in the chronic CMS model, substantiating our recent study with an acute depression model^12^. Furthermore, auricular ECS was associated with adverse effects that were significantly less often or not at all observed with cortical ECS, which we therefore consider a more valid procedure to mimic ECT. As cortical ECS has higher construct validity than auricular ECS (because, as in patients, cortical ECS stimulates the cerebral cortex and not the brain stem as auricular ECS), we assume that cortical ECS as an antidepressive treatment in the CMS model has a higher translational value than auricular ECS. Furthermore, the significant adverse effects (fear calls, weight loss, and transient motor paralysis) associated with auricular ECS^12^ raise ethical concerns against this method, whereas tolerability of cortical ECS in rats is much better.

After establishing cortical ECS as an effective treatment to reduce depression-like symptoms in the CMS model, the major aim of our study was to identify potential biomarkers for ECT response. Considerable experimental evidence implicates p11 in the mechanism of action of antidepressant drugs and ECS, in part due to its interaction with BDNF and specific serotonin (5-HT) and glutamate receptors^15,31^, so that our hypothesis was that ECT responders differ from nonresponders in respect to p11 expression or epigenetic regulation. Indeed, a higher p11 mRNA expression was found in PFC of ECS responders, which was paralleled by a significantly increased BDNF expression, which, however, was also significantly increased in ECS nonresponders. Furthermore, p11 promoter methylation was increased in PFC of ECS responders, whereas BDNF promoter methylation was not altered. These findings prompted us to perform a clinical POC study focusing on peripheral p11 promoter methylation in pharmacoresistant MDD patients that respond or do not respond to ECT. In this prospective (longitudinal) study, we found higher p11 promoter methylation levels in patients who responded to ECT. The observed differences were present at baseline and during the ECT course, although there were no acute changes in peripheral p11 promoter methylation after treatment. Most importantly, we were able to replicate this finding in an independent cohort of 65 depressed patients receiving ECT.

To our knowledge, this is the first study that, based on both preclinical and clinical evidence, indicates that p11 methylation may be a suitable biomarker of ECT response in MDD. p11 levels are regulated by BDNF^32^ and several previous studies have investigated whether serum BDNF could be a biomarker for therapy response to ECT, but inconsistent results were obtained^24,33^. Thus, as indicated by a recent systemic review and meta-analysis, ECT can enhance serum BDNF levels, but this effect does not necessarily correlate with its clinical response in depression^33,34^. The complex regulation of BDNF expression underlies epigenetic mechanisms, such as histone modification and DNA methylation^35^. With respect to ECT response, we recently found that BDNF promoter methylation rates, especially concerning the exon I promoter, may be more suited than BDNF serum levels to predict an antidepressant effect of ECT in pharmacoresistant MDD patients^22^.

Behavioral studies demonstrated that p11 knockout mice are insensitive to the antidepressant actions of BDNF^32^. Only two studies, to our knowledge, have investigated p11 as a biomarker for responsiveness to antidepressant treatments^36,37^. In one study, no differences between responders and nonresponders to therapy with escitalopram or nortriptyline were reported for leukocyte mRNA levels of p11^36^, whereas the other study found that MDD patients that responded to citalopram had lower p11 levels in natural killer cells and monocytes than nonresponders^37^. As noted by the authors, it appears paradoxical that a decrease of p11 in white blood cells should be associated with antidepressant response, as animal studies have shown that antidepressants increase p11 in the brain^15^. Melas et al.^38^ reported that this increase of p11 after administration of escitalopram was accompanied by a hypomethylation within the p11 promoter region in the PFC of the Flinders Sensitive Line genetic rodent model of depression.

In contrast to the study by Melas et al.^38^, we observed higher p11 expression accompanied by higher p11 promoter methylation in the PFC of responders to ECS. In our clinical sample we observed higher p11 promoter methylation in blood samples of responders to ECT.

The positive correlation between p11 promoter methylation and p11 expression is in line with recent data from our laboratory showing a positive correlation of p11 methylation and transcriptional activity in the hippocampus and PFC of rats^13^.Moreover, there is growing evidence that the function of CpG methylation is not necessarily gene silencing, for example due to hypermethylation of repressive elements^39,40^. Probably, the observed methylation differences in responders are not specific to p11 but to other genes with a similar regulation pattern. The difference between our results and the ones reported by Melas et al.^38^ could also be explained by the difference in the measured p11 sequence, as our fragment also covered the regulatory region of exon I near the p11 promoter. Furthermore, we already showed that different substrains of rats respond differently to CMS, reflected by different p11 methylation and expression^13^.

Nevertheless, two studies on the epigenetic regulation of the p11 gene showed a lower expression in association with a hypermethylation of the p11 promoter region, one in primary human pituitary tumors and another in medulloblastoma primary tumors and cell lines^41,42^. Thus, ECS-induced hyperexpression could also have caused p11 hypermethylation as a regulating feedback loop.

Our study is restricted in its ability to understand the pathophysiology behind the observed differences in p11 methylation. Nevertheless, one could speculate that p11 functionality at baseline is necessary for ECT to be fully effective or that our findings point to a subgroup of depressed patients, especially sensitive to ECT. Possibly, higher p11 expression and/or methylation at baseline is favorable for the ECT-induced neurogenesis via BDNF. In this regard, it is interesting to note that p11 activates tissue plasminogen activator, which is involved in the cleavage of proBDNF to BDNF^43^. Furthermore, the BDNF-induced neural plasticity has been proposed to be dependent on p11 in a cell culture model^44^.

Alternatively, the higher p11 methylation in responders could point to a subgroup of patients in whose p11 hypermethylation (and possibly low expression of p11) indicate a trait of MDD sensitive to ECT.

Our study has several limitations. These include the fact that we did not assess p11 or BDNF methylation or mRNA in the peripheral circulation of rats. Furthermore, due to technical reasons, we were not able to assess p11 mRNA or BDNF mRNA in the samples collected from patients. Thus, we can only speculate about the expression of p11 in the clinical cohort and future studies are needed to understand the regulatory mechanisms of p11 methylation to obtain a more mechanistic insight. In addition, we cannot exclude differences in peripheral cell composition of responders and nonresponders that could have influenced our methylation results. Furthermore, we performed no single-nucleotide polymorphism analysis in the p11 gene, which could probably have influenced p11 methylation. Another limitation is our highly selective patient cohort, which included only severely affected and medicated patients. Thus, we were not able to control for medication and future studies are needed to clarify the specificity of response prediction of p11 methylation regarding different treatment options.

In conclusion, we have taken a novel approach to studying whether p11 or its epigenetic regulation is a potential biomarker of response to ECS/ECT. First, we modified a widely used rat model of chronic depression, CMS, by using a rat substrain that is particularly stress-sensitive, and, as shown here, resistant to the antidepressant citalopram. Next, ECS was shown to be more effective than citalopram in this model, thus allowing to select ECS responders and nonresponders. The response to ECS was correlated with p11 mRNA expression and methylation in PFC, which prompted a POC clinical pilot trial in patients with refractory MDD, showing higher p11 promoter methylation in remitters to ECT. In an independent replication sample acquired in a different university hospital by independent researchers, we were able to replicate these differences in baseline p11 methylation and tested the predictive properties of p11. We found that patients with a p11 methylation below a threshold with highest sensitivity and specificity had a fourfold increased risk for nonresponse to treatment, whereas in the same sample elevated p11 methylation predicted response to ECT with positive predictive value of around 90%. Using the same thresholds in the POC sample, we found that all responders had p11 hypermethylation above the threshold, indicating that p11 promoter methylation is a suitable biomarker for response to ECT.

Within the last years, increasing efforts have been made to search for epigenetic biomarkers of response to pharmacological antidepressant treatments. Regarding ECT, there is only limited data on epigenetic effects of ECT in general. Up to date, our previous study on BDNF methylation^22^ is the only investigation on epigenetic alterations of a candidate gene as a possible biomarker for response to ECT. Furthermore, most of the studies on ECS in rodent models do not distinguish between response types and thus have only limited translational value to identify possible biomarkers.

We show for the first time that a translationally and clinically valid biomarker—p11 methylation—can distinguish between responders and nonresponders of ECT treatment before the first treatment is initiated, which allows for an individualization of treatment strategies in pharmacoresistant depression. Patients likely to respond to ECT should receive ECT treatment more easily and probably more early in the course of the disorder. Patients likely not responding to ECT should not receive ECT treatment, which will lead to increased acceptance of this still stigmatized treatment option. Further studies are needed to elucidate additional markers increasing the predictive power to distinguish between responders and nonresponders to ECT, and to identify other treatment options effective for ECT-resistant patients.

## Electronic supplementary material

Supplemental Information_clean version

## References

[1] Hasler G (2010). Pathophysiology of depression: do we have any solid evidence of interest to clinicians?. World Psychiatry.

[2] Gold PW, Machado-Vieira R, Pavlatou MG (2015). Clinical and biochemical manifestations of depression: relation to the neurobiology of stress. Neural Plast..

[3] Rush AJ, Thase ME, Dube S (2003). Research issues in the study of difficult-to-treat depression. Biol. Psychiatry.

[4] Bennabi D (2015). Risk factors for treatment resistance in unipolar depression: a systematic review. J. Affect. Disord..

[5] Lisanby SH (2007). Electroconvulsive therapy for depression. N. Engl. J. Med..

[6] Scott AI (2010). Electroconvulsive therapy, practice and evidence. Br. J. Psychiatry.

[7] Brown ED, Lee H, Scott D, Cummings GG (2014). Efficacy of continuation/maintenance electroconvulsive therapy for the prevention of recurrence of a major depressive episode in adults with unipolar depression: a systematic review. J. Ect..

[8] Nordenskjold A, von Knorring L, Engstrom I (2012). Predictors of the short-term responder rate of electroconvulsive therapy in depressive disorders--a population based study. BMC Psychiatry.

[9] Haq AU, Sitzmann AF, Goldman ML, Maixner DF, Mickey BJ (2015). Response of depression to electroconvulsive therapy: a meta-analysis of clinical predictors. J. Clin. Psychiatry.

[10] Kendler KS, Karkowski LM, Prescott CA (1999). Causal relationship between stressful life events and the onset of major depression. Am. J. Psychiatry.

[11] Willner P, Muscat R, Papp M (1992). Chronic mild stress-induced anhedonia: a realistic animal model of depression. Neurosci. Biobehav. Rev..

[12] Theilmann W (2014). A new method to model electroconvulsive therapy in rats with increased construct validity and enhanced translational value. J. Psychiatr. Res..

[13] Theilmann W (2016). Behavioral differences of male Wistar rats from different vendors in vulnerability and resilience to chronic mild stress are reflected in epigenetic regulation and expression of *p11*. Brain Res..

[14] Rescher U, Gerke V (2008). S100A10/p11: family, friends and functions. Pflug. Arch..

[15] Svenningsson P, Kim Y, Warner-Schmidt J, Oh YS, Greengard P (2013). p11 and its role in depression and therapeutic responses to antidepressants. Nat. Rev. Neurosci..

[16] Svenningsson P (2006). Alterations in 5-HT1B receptor function by p11 in depression-like states. Science.

[17] Duman RS, Heninger GR, Nestler EJ (1997). A molecular and cellular theory of depression. Arch. Gen. Psychiatry.

[18] Castren E, Voikar V, Rantamaki T (2007). Role of neurotrophic factors in depression. Curr. Opin. Pharmacol..

[19] Duman RS, Monteggia LM (2006). A neurotrophic model for stress-related mood disorders. Biol. Psychiatry.

[20] Duclot F, Kabbaj M (2015). Epigenetic mechanisms underlying the role of brain-derived neurotrophic factor in depression and response to antidepressants. J. Exp. Biol..

[21] Bjorkholm C, Monteggia LM (2016). BDNF e a key transducer of antidepressant effects. Neuropharmacology.

[22] Kleimann A (2015). BDNF serum levels and promoter methylation of BDNF exon I, IV and VI in depressed patients receiving electroconvulsive therapy. J. Neural Transm. (Vienna).

[23] Christensen T, Bisgaard CF, Wiborg O (2011). Biomarkers of anhedonic-like behavior, antidepressant drug refraction, and stress resilience in a rat model of depression. Neuroscience.

[24] Willner P, Towell A, Sampson D, Sophokleous S, Muscat R (1987). Reduction of sucrose preference by chronic unpredictable mild stress, and its restoration by a tricyclic antidepressant. Psychopharmacol. (Berl.).

[25] Bergstrom A, Jayatissa MN, Thykjaer T, Wiborg O (2007). Molecular pathways associated with stress resilience and drug resistance in the chronic mild stress rat model of depression: a gene expression study. J. Mol. Neurosci..

[26] Bisgaard CF (2007). Proteomic investigation of the ventral rat hippocampus links DRP-2 to escitalopram treatment resistance and SNAP to stress resilience in the chronic mild stress model of depression. J. Mol. Neurosci..

[27] Willner P (2005). Chronic mild stress (CMS) revisited: consistency and behavioural-neurobiological concordance in the effects of CMS. Neuropsychobiology.

[28] Nieto-Gonzalez JL (2015). Presynaptic plasticity as a hallmark of rat stress susceptibility and antidepressant response. PLoS. ONE.

[29] Moreau JL, Scherschlicht R, Jenck F, Martin JR (1995). Chronic mild stress-induced anhedonia model of depression; sleep abnormalities and curative effects of electroshock treatment. Behav. Pharmacol..

[30] Henningsen K, Woldbye DP, Wiborg O (2013). Electroconvulsive stimulation reverses anhedonia and cognitive impairments in rats exposed to chronic mild stress. Eur. Neuropsychopharmacol..

[31] Lee KW (2015). Alteration by p11 of mGluR5 localization regulates depression-like behaviors. Mol. Psychiatry.

[32] Warner-Schmidt JL (2010). A role for p11 in the antidepressant action of brain-derived neurotrophic factor. Biol. Psychiatry.

[33] Brunoni AR, Baeken C, Machado-Vieira R, Gattaz WF, Vanderhasselt MA (2015). BDNF blood levels after non-invasive brain stimulation interventions in major depressive disorder: a systematic review and meta-analysis. World J. Biol. Psychiatry.

[34] Polyakova M (2015). Brain-derived neurotrophic factor and antidepressive effect of electroconvulsive therapy: systematic review and meta-analyses of the preclinical and clinical literature. PLoS. ONE.

[35] Boulle F (2012). Epigenetic regulation of the BDNF gene: implications for psychiatric disorders. Mol. Psychiatry.

[36] Cattaneo A (2013). Candidate genes expression profile associated with antidepressants response in the GENDEP study: differentiating between baseline ‘predictors’ and longitudinal ‘targets’. Neuropsychopharmacology.

[37] Svenningsson P (2014). Preliminary evidence that early reduction in p11 levels in natural killer cells and monocytes predicts the likelihood of antidepressant response to chronic citalopram. Mol. Psychiatry.

[38] Melas PA (2012). Antidepressant treatment is associated with epigenetic alterations in the promoter of P11 in a genetic model of depression. Int. J. Neuropsychopharmacol..

[39] Hellman A, Chess A (2007). Gene body-specific methylation on the active X chromosome. Science.

[40] Jones PA (2012). Functions of DNA methylation: islands, start sites, gene bodies and beyond. Nat. Rev. Genet..

[41] Lindsey JC (2007). Epigenetic deregulation of multiple S100 gene family members by differential hypomethylation and hypermethylation events in medulloblastoma. Br. J. Cancer.

[42] Dudley KJ, Revill K, Whitby P, Clayton RN, Farrell WE (2008). Genome-wide analysis in a murine Dnmt1 knockdown model identifies epigenetically silenced genes in primary human pituitary tumors. Mol. Cancer Res..

[43] Tsai SJ (2007). The P11, tPA/plasminogen system and brain-derived neurotrophic factor: implications for the pathogenesis of major depression and the therapeutic mechanism of antidepressants. Med. Hypotheses.

[44] Park SW (2016). p11 mediates the BDNF-protective effects in dendritic outgrowth and spine formation in B27-deprived primary hippocampal cells. J. Affect. Disord..

