# Supplementary material for: P11 promoter methylation predicts the antidepressant effect of electroconvulsive therapy
Source: Transl Psychiatry. 2018 Jan 22;8:25. doi: 10.1038/s41398-017-0077-3 (PMC5802592; doi:10.1038/s41398-017-0077-3)
Supplement: Supplementary file 1 — Supplemental Information_clean version [file 41398_2017_77_MOESM1_ESM.docx]

**P11 promoter methylation predicts the antidepressant effect of electroconvulsive therapy**

***Supplemental Information***

**Supplemental Methods**

Driven by findings from our previously established animal model of ECT with an enhanced translational value (1) and our observations of breeder-related differences in behavior and p11 methylation (2), this study aimed to explore the translational value of p11 promoter methylation as a biomarker for response to electroconvulsive stimulation. BDNF was included for comparison.

**A. Preclinical experiments**

***Animals***

Altogether 46 male Wistar outbred rats (Crl:WI(Han); 7-8 weeks of age; Charles River, Sulzfeld, Germany) underwent CMS-procedure as described below. Rats were singly-housed in Makrolon type III cages (Ebeco, Castrop-Rauxel). Additional 10 age-matched male Wistar rats were purchased from Charles River to serve as unstressed controls. Rats were singly-housed in Makrolon type III cages (Ebeco, Castrop-Rauxel, Germany). Fifteen adult male Wistar rats, which were purchased at an age of 8 weeks from Janvier (Saint-Berthevin, France) and housed in groups of four rats in Makrolon type IV cages, were used as unstressed test partners for social interaction test. Age at social interaction was 15 weeks for experiment 1 and 30 weeks for experiment 2 (see below). Standard laboratory chow (Altromin 1324 standard diet; Altromin, Lage, Germany) and tap water were provided *ad libitum*, except when CMS procedure required short-term food or water deprivation. The controlled 12h light/12h dark schedule was only disturbed during stress procedure. All rats were adapted to the laboratory and habituated to handling for at least one week before starting the experiments. Group allocation in animal experiments was performed randomly by drawing lots. Experiments were performed according to the EU council directive 210/63/EU and were approved by the animal subjects review board of our institution (# 12/0871). All efforts were made to minimize pain or discomfort of the animals used.

***Chronic mild stress (CMS) protocol***

We have previously shown that outbred rats from Charles River (Sulzfeld, Germany), including the Wistar and Sprague-Dawley strains, exhibit increased anxiety-related behavior and increased response to CMS compared to such rats from other breeders, which could be either a result of genetic divergence between outbred subpopulations or of vendor-related differences in housing and handling conditions (2,3). Thus, we expected that CMS-induced behavioral alterations induced in the substrain of Wistar outbred rats (Crl:WI(Han)) from Charles River used for the present experiments might be particularly severe and difficult to treat by antidepressants. The CMS protocol was derived from that described by Willner et al. (4). Briefly, following mild stresses were randomly applied every day, over a period of 3 weeks: social crowding (four rats were placed in one Makrolon type III cage for 7 h), swimming in cold (15°C) or hot water (40°C) for 5 or 10 min, wet bedding for 16 h, food deprivation for 21.5-24 h, water deprivation for 14-21 h, restraint stress for 0.5-1 h and continuous light for 36 h.

***Behavioral tests***

Before, during and after the CMS protocol, a battery of behavioral tests was performed to characterize the depression-like profile of the rats (Fig. 1). As a result of technical reasons, the experimenter was not blinded to animal experiments with the exception of forced swim test evaluation.

***Sucrose consumption test (SCT).*** Hedonic deficits induced by CMS can be measured as a decrease in consumption or preference of sweetened solution (4,5). In the present study, we used the SCT, in which singly housed rats had free access for a 14 h overnight period to a bottle of 1% sucrose solution and a bottle of tap water. The amount of consumed sucrose solution was measured in [g] by weighing the bottles. No previous food or water deprivation was provided to avoid metabolic influences or acute stress before testing. The position of the bottles was switched after each test session to avoid any place preference. As shown in Figure 1, 3-4 SCT habituation sessions were performed before CMS, and the mean sucrose intake of the last three habituation sessions was defined as basal value (100%). During CMS, SCT was performed once a week (Fig. 1) and the individual percentage change of sucrose intake was estimated.

After three weeks of CMS, rats were classified as anhedonic-like or hedonic-like, based on changes in intake of sucrose solution. According to Christensen et al. (6), anhedonic-like rats are supposed to show a >25% within-subject decrease in sucrose consumption. Hedonic-like rats are supposed to show a <10% within-subject decrease in sucrose consumption. Animals not responding to either criterion are considered as unclassifiable.

***Forced swim test.*** The forced swim test was performed according to Porsolt et al. (7). Rodents were individually placed in a transparent plexiglas cylinder (50 cm deep, 25 cm diameter) containing 20 cm of water (25 ± 1 °C). At the first day of CMS, a 5 min pre-test trial was performed followed by a 5 min test trial 20 days later. The retest trial (5 min) was performed 30 h after the fifth electroconvulsive stimulation (ECS; see below). Behavior was recorded with a HD-camcorder (Canon Legria HFS21) and the immobility time (making only those movements necessary to keep the head above the water) for each rat during the trials was quantified.

***Open field test (OFT).*** The open field test (8) is a routine method to measure locomotor activity and anxiety like behavior. The test was performed in a round open field made of black PVC (diameter 80 cm, height 80 cm), which was divided virtually into three zones (center, inner zone, outer zone). The animals were placed individually in the center of the open field. Distance moved [cm] and time spent in the center of the open field [sec] was recorded for 5 min and analyzed with EthoVision®XT7 software (Noldus Information Technology, Wageningen, Netherlands).

***Novelty-induced hypophagia.*** The suppression of appetitive behavior induced by novelty provides an anxiety related measure (9,10), which is normalized with chronic, but not short-term, antidepressant drug treatment (11). Prior to testing the rats were habituated to consume palatable Leibnitz Minis butter biscuits (Bahlsen, Hannover, Germany). During habituation sessions biscuits were offered to the rats in their home cages for altogether six times and the animals were monitored to ensure the preferring of the biscuits. No prior food or water deprivation was applied. A weighing pan with two quartered biscuits was stuck to the center of a round open field made of light grey PVC (diameter 80 cm, height 25 cm). Animals were placed singly at the edge of the open field with view towards the center. The latency to eat [sec] (time point, at which the rat began to chew continuously) and the total amount of eaten biscuits [g] was estimated manually for 5 min. For pretest and test session the same open field was used, because preliminary experiments with different cohorts of rats revealed that naïve rats did not adapt to the open field within one session and that the measured parameters were similar in pretest and test in the same open field.

***Social interaction test.*** The social interaction test assesses anxiety-related behavior (12) and was performed in a round open field made of black PVC (diameter 80 cm, height 25 cm). Naïve male Wistar rats (Janvier), which were approximately equal in weight and age, and which were unknown to the stressed animals, were used as test partners. The animals were marked with varicolored animal marking sticks (Raidex, Dettingen/Erms, Germany) and placed individually in the center of the open field. Mean distance between subjects and the time spent in close body contact (distance between nose-nose and nose-tail ≤4 cm) was recorded for 5 min and analyzed with EthoVision®XT7 software (Noldus).

***Weight measurement.*** Body weight was determined during the whole course of experiments as a measure for the general condition. The weight gain of the ECS-treated rats during CMS-exposure was compared to average weight development of naïve male Wistar rats at the same age (11-14 weeks), which is approximately 15 g per week (Charles River) concerning breeder’s declaration and experience drawn from previous investigations. A reduction in body weight or a diminished weight gain point to a reduced well-being of the rats (Morton and Griffiths, 1985).

***Antidepressive treatments***

Two preclinical experiments were performed as illustrated in Figure 1. In experiment I, we studied whether prolonged treatment with the antidepressant drug citalopram affected the CMS-induced alterations in the SCT. In experiment II, we compared two types of ECS, auricular and cortical ECS, in CMS-exposed rats. We have previously shown in acute models of depression that cortical ECS is more effective and has higher construct validity than auricular ECS (1). In both experiments, the animals were randomly divided into the treatment groups (based on pretreatment SCT data) and it was ensured that the numbers of anhedonic-like, hedonic-like and unclassifiable rats were almost the same in all treatment groups (cf., Table S3).

***Experiment I.*** Sixteen rats were exposed to CMS for 21 days before drug treatment started (Fig. 1A). Rats were randomly assigned to treatment groups, vehicle (0.9% saline; *n*=8) and citalopram (*n*=8) and treated for 34 days while stress exposure was continued. Citalopram (Cipramil®, Lundbeck, Denmark) was administered i.p. once daily in the morning in a dosage of 15 mg/kg; the dosage was chosen based on Kusmider et al. (13). Altogether stress exposure lasted for 55 days. Baseline sucrose consumption and body weight were measured before CMS. Moreover, habituation to palatable food required for novelty-induced hypophagia was performed before starting the stress procedure. During CMS, sucrose consumption and body weight was measured once per week. Finally, rats performed the open field test, novelty-induced hypophagia test and social interaction test. Rats were decapitated 24 h after the final drug injection. One rat was excluded from the experiment due to citalopram injection-induced necrosis. Treatment groups were allocated randomly by drawing of lots. Due to technical reasons, the experimenter was not blinded during the experiments.

***Experiment II.*** Before CMS treatment, 30 rats were implanted under brief isoflurane anesthesia with cortical screw electrodes (placed bilaterally above the frontal cortex) as described recently (1). These electrodes served to record the electroencephalogram (EEG) and to deliver ECS in rats with cortical ECS. One rat died after surgery, so that total group size of these experiments was 29. To avoid any effects of anesthesia on subsequent induction of behavioral alterations, the interval between surgery and onset of CMS was at least 2 weeks (Fig. 1B).

Baseline sucrose consumption and body weight was measured before CMS. Moreover, habituation to palatable food required for novelty-induced hypophagia was performed before starting the stress procedure. During CMS, sucrose consumption and body weight were measured once per week. After three weeks of stress, rats performed the forced swim test and novelty-induced hypophagia test. Afterwards, rats were randomly assigned to treatment groups: sham ECS (*n*=9), cortical ECS (*n*=10) and auricular ECS (*n*=10). Rats received ECS or sham-ECS treatments once daily for five consecutive days. Afterwards, rats were tested in sucrose consumption, open field, forced swim test, novelty-induced hypophagia and social interaction test. After further two ECS treatments, rats were decapitated (Fig. 1B). Due to technical reasons one sham animal and one ECS positive responder were excluded from statistical analyses of BDNF expression. These criteria were not pre-established. Treatment groups were allocated randomly by drawing of lots. Due to technical reasons, the experimenter was not blinded during the animal experiments with the exception of the forced swim test evaluation.

For auricular ECS the stimulus was administered via ear-clip electrodes using the ECT Unit 57800 device (Ugo Basile, Comerio, Italy). Cortical ECS was performed via the two frontal screw electrodes using the A310 Accupulser (World Precision Instruments, Sarasota, USA). The stimulus consisted of bidirectionally applied square wave pulses. Stimulation parameters for auricular ECS used in this study were 0.9 ms pulse-width, 100 pulses/s, 0.5 s duration, 75-85 mA. This corresponds to a charge of 6.75-7.65 mC. Parameters for cortical ECS were 1 ms pulse-width, 100 pulses/s, 1 s duration, 6.5-9 mA, which corresponds to a charge of 1.3-1.8 mC. The stimulus parameters were based on our previous study (1) to induce generalized convulsive seizures of at least 15 sec duration (see Results). For EEG recordings a one-channel amplifier (ADinstruments Ltd., Sydney, Australia) and an analog-digital converter (PowerLab/800s, ADinstruments) were used. Further, the seizure type was determined. Sham animals underwent the same handling procedure without electrical stimulation. For sham treatment, rats were connected with ear-clip electrodes and conduction cable, but did not receive electrical stimulation.

As recently reported (1), ultrasonic vocalization was recorded via Avisoft recorder (version 3.4.2, Avisoft Bioacoustic, Berlin, Germany) for 5 min during most of the ECS sessions, starting one minute before stimulation. Number and duration of 22 kHz-calls, which are associated with distress and fear of rats (duration: >20 ms; frequency: 18-32 kHz)(*54*), were analyzed.

***Classification of treatment response.*** After antidepressant treatment, alterations in anhedonic-like behavior were assessed by estimating within subject changes in sucrose consumption. According to Christensen et al. (6), positive treatment responders were anhedonic-like animals showing a >20% within-subject increase in sucrose consumption, whereas nonresponders were rats showing a <20% within-subject increase in sucrose consumption. Moreover, treatment-induced decrease in sucrose consumption of >50% of previous anhedonic-, hedonic-like, or unclassifiable rats was characterized as negative response.

Next to anhedonic-like behavior, additional depressive- and anxiety-like symptoms were estimated (cf., Table S4). Altogether nine symptom items (sub-categories), summarized in five symptom groups, were characterized after treatment. As additional tenth symptom, behavioral despair of (sham-) ECS-treated rats was estimated in the forced swim test (Table S4). For each symptom item, rats were characterized individually as sub-categorical positive responders, sub-categorical nonresponders or sub-categorical negative responders. The criterion for “sub-categorical positive responders“ and “sub-categorical negative responders“ was defined as performance of one standard deviation unit below or above the mean of sham or vehicle treated animals. All rats that were within one standard deviation of the mean of vehicle or sham-ECS treated animals were considered as “sub-categorical non-responders”.

***Brain tissue processing***

Rats were anesthetized with carbon dioxide and decapitated 24 h after the last (sham)-ECS, citalopram or vehicle treatment. Brains were removed, PFCs were dissected and stored at -80°C until further processing (see below). The PFC was chosen for p11 analyses because it is thought to be a critical neural substrate for depression (14) and is involved in p11-mediated antidepressant effects in rodent models (15,16).

**B. Clinical experiments**

***Proof-of-concept (POC) study***

We conducted a prospective study, including eleven patients suffering from pharmacoresistant major depressive disorder (MDD). Patients’ characteristics are shown in Table S1. Diagnoses were established using the German version of the Structured Clinical Interview for DSM IV diagnoses. Depression severity was assessed before ECT treatment session 1, 4, 7 and 10 using the Montgomery Asberg Depression Scale (MADRS)(17). A ≥50 % reduction of the MADRS score was interpreted as response to therapy.

ECT was applied in three sessions per week as common practice in the facility with a customized Thymatron IV brief-pulse device (Somatics; Lake Bluff, IL, USA). Motor and EEG seizure duration was monitored and stimulus intensity was adjusted accordingly.

The study adhered to the Declaration of Helsinki (1964) and its later amendments. It was approved by the Ethics Committee of the University of Erlangen (3252/2006). Written informed consent was obtained from all patients after the procedures had been fully explained to them and prior to their inclusion in the study. All patients were recruited as in-patients at the Department of Psychiatry and Psychotherapy of the University Hospital Erlangen. Fasting blood samples were taken directly before (8–10 a.m.), ECT sessions 1, 4, 7 and 10. All blood samples were stored at -80 °C immediately after collection.

***Independent replication sample***

Following the POC study, an independent replication sample was acquired in a different university hospital by other researchers to assure a blinded performance of the molecular analyses. For this replication sample, 67 patients with pharmacoresistant major depressive disorder and ECT were enrolled at the Department of Psychiatry of the Charité Berlin; from this group, fasting blood samples from 65 patients with ECT were available. Patients’ characteristics are shown in Table S2. Patients enrolled were eligible for inclusion if they were 18 to 85 years old and met Structured Clinical Interview for DSM-IV criteria for unipolar depression with a pretreatment 24-item Hamilton Rating Scale for Depression (HRSD-24) score of >18, ability to provide written informed consent, and an indication for ECT as indicated by an independent psychiatrist. Main exclusion criteria were a diagnosis of schizophrenia, schizoaffective or bipolar disorder, dementia, delirium or other central nervous system disease with the probability of affecting cognition or response to treatment, substance dependence within the past year, or ECT within the past 6 months before acute phase.

Depression severity was assessed before and weekly (6 weeks) during ECT-treatment using the Hamilton Depression Scale (18). A ≥50 % reduction of the HAMD score within the treatment period was interpreted as response to therapy.

Right unilateral ECT was administered three times a week with an ultra-brief pulse device with pulse lengths of 0.3 milliseconds (Mecta 5000Q, Somatics, Belleville, Illinois). Anesthesia was performed with propofol (approximately 1.5 mg/kg/body weight) or etomidate (approximately 0.75 mg/kg/ body weight) and succinylcholine (approximately 0.75 mg/kg/body weight) for relaxation. Motor and electroencephalogram seizure duration were monitored to control for adequate duration. Seizure threshold was titrated during the first treatment and energy was only increased if patients did not respond clinically or showed insufficient seizures during the ECT course (i.e., motor response of >20 sec or EEG seizure activity of >30 sec). EEG was recorded from two channels using frontomastoid placements and monitored during ECT to confirm seizure activity and to document seizure duration. Bilateral ECT was started in patients without improvement in 2 consecutive weeks and terminated after six treatment sessions without clinical improvement.

The study adhered to the Declaration of Helsinki (1964) and its later amendments. It was approved by the Ethics Committee of the Charité Berlin (EK-224-05c). Written informed consent was obtained from all patients after the procedures had been fully explained to them and prior to their inclusion in the study. Fasting blood samples were taken once before the initation of the ECT treatment series.

**C. Biochemical and molecular analyses**

***Tissue and blood processing*.** All procedures for analysis of p11 mRNA expression and p11/BDNF methylation have been described in more detail elsewhere (2,19,20). In brief, DNA and RNA were purified using peqGOLD TriFast (Peqlab, Erlangen, Germany) for brain tissue and QIAmp DNA Blood Mini Kit (QIAGEN GmvH, Hilden, Germany) for EDTA blood.

***mRNA expression by quantitative real-time PCR.*** RNA concentrations were determined with the NanoDrop ND-1000 Spectrophotometer (NanoDrop Technologies Inc., USA). Afterwards approximately 500ng of cDNA were synthesized by reverse transcription of RNA using the iScript™ cDNA Synthesis Kit (Bio-Rad Laboratories, Hercules, CA). cDNA samples were kept at -80 °C until PCR analysis. Quantative PCR was performed with 500ng of cDNA in triplicates of each sample and NTC´s (no template controls) using the GoTaq qPCR Master Mix (Promega). P11 mRNA expression was measured as main target along with GAPDH and β-actin as housekeeping genes (primers shown in Table S5). Efficiency of all primers was tested with a dilution series (all primers between 0.95-1). Data were obtained as threshold cycle (Ct) values and analyzed using the Biogazelle’s qbase+ 2.0 software. Relative expression levels were normalized with respect to housekeeping genes, experimental and technical errors. Quality control settings excluded wells with too high/low Cq or samples with high replicate variability. Relative expression quantification analysis was performed with the qBase method, which has been shown to be an advanced model compared to the classic delta-delta-Ct method (21).

***DNA methylation assay.*** The isolated genomic DNA was cleaned up by NucleoMag® Blood 200 µl (Macherey-Nagel, Düren, Germany). Afterwards, DNA samples were bisulfite-converted and purified by the EpiTect® 96 Bisulfite Kit (QIAGEN AG) according to the manufacturer’s recommendations. DNA was amplified through (semi-) nested touch-down PCR. Primer sets for amplification of the rat p11 and BDNF and the human p11 promoter region (Metabion GmbH, Steinkirchen, Germany) are listed in Tables S6-S8; target sequences are given in Supplemental figures S6-S8. Bisulfite-treated samples were purified via Agencourt® AMPure® XP magnetic beads (Beckman Coulter, Krefeld, Germany) and sequenced using the reverse primer via by BigDye® Terminator v3.1 Cycle Sequencing Kit (Applied Biosystems, Foster City, CA, USA) and an Applied Biosystems/HITACHI 3500xl Genetic Analyzer (Applied Biosystems). Sequences and electropherograms were analyzed via the specialized Epigenetic Sequencing Methylation (ESME) analysis software (22) and the percentage methylation of each CpG site within the amplified region was estimated by the ratio between peak values of Cytosine (C) and Thymine (T) (C/T). An exemplary ESME analysis of the human P11 promoter sequence is provided in Supplemental figure S9. The epigenetic sequencing methylation analysis software (ESME) corrects for incomplete bisulfite conversion and performs quality control tests on data before mapping the methylation rates/positions to the reference sequence. All obtained sequences were screened for sequencing quality using ABI sequence scanner (Applied Biosystems). Samples with a QV-value <20 were measured again. For the final analysis, all sequences were above the QV threshold of 20. With our established methods we are able to detect differences in methylation rate per CpG around 5%.

***BDNF protein analyses.*** For total protein extractions, the brain tissue samples were homogenized in cold 1% NP-40 lysis buffer as described (23). BDNF protein levels were analyzed using a commercial ELISA kit and western blotting. For the BDNF immunoassay (BDNF Emax Immunoassay System, Promega, Madison, WI, USA), recombinant human mature BDNF protein (Peprotech, USA) and transiently acidified samples were 1:5 diluted with Block and Sample buffer and transferred to 96-well Maxisorb plates (Nunc, USA) pre-blocked and pre-coated with the BDNF antibody (in carbonate buffer pH 9.7). Following 2 h incubation at room temperature the plates were washed with TBS-T and incubated with secondary BDNF antibody for another 2 h. After subsequent wash, the plates were incubated with HRP-conjugated IgY antibody directed against the secondary antibody. TMB substrate was used to initiate enzymatic reaction, which was stopped within 15 min with 1N HCl. Absorbance was measured at 450 nm. BDNF content was calculated according to the standard curve and normalized against total protein of the samples. The specificity of the analysis was validated using hippocampal samples obtained from BDNF^-/-^ mouse (kindly provided by Dr. Eero Castrén, Helsinki, Finland).

**Statistical analysis**

Based on previous experiments with ECS, CMS, and SCT (1,2) and preliminary experiments, the sample size of the preclinical experiments was chosen to ensure adequate power (1-β=0.8; α=0.05) for detection of significant ECS effects. Depending on whether data were normally distributed or not, either parametric or nonparametric tests were used for statistical evaluation of preclinical data as explained in figure legends. Brown-Forsythe tests were applied to control whether variances were equal between groups. For all comparisons, the assumption of equal variances was true. Fisher´s exact test was used to compare the occurrence of anhedonic-like behavior in rats after CMS-exposure with unstressed rats. For statistical analyses of data from three groups of rats, one-way ANOVA or Kruskal-Wallis test with *posthoc* Dunn’s or Dunnett’s multiple comparison tests were used except for analysis of p11 mRNA expression, which was performed using mixed linear models for repeated measurements (to include technical replicates from qPCR) followed by Sidak’s *posthoc* test and for analysis of p11 methylation, which was performed using mixed linear models for repeated measurements (CpG was used as repeated variable in the analyses). Accordingly, analyses of p11 promoter methylation in both patient cohorts was performed using mixed linear models for repeated measurements including the factors age and gender (24).

Based on the findings from the clinical POC trial we calculated the sample size for the replication study. With the mean differences and standard deviation found in the POC trial and also the sampling ratio of responders/nonresponders (4/7 = 0.571) a sample size in the nonresponder group of 14 patients (and 8 patients responding respectively) would be necessary to detect a true difference of the same size (Power: 1-β=0.8; α=0.05). In the replication sample, we indeed found 15 patients that did not respond to treatment, while the sampling ratio was in this case 3.33. Next, receiver operating characteristic (ROC) analyses were established for the replication sample to determine the optimal p11 promoter methylation able to discriminate responders from nonresponders to ECT. The optimal cutoff threshold values were determined at the point on the ROC curve at which the Youden´s Index (sensitivity + specificity−1) was maximal. Last, we applied the obtained Youden´s Index to our POC study. Fisher´s exact test was used to compare the p11 methylation level cutoff between responders and nonresponders in both patient cohorts. Bonferroni corrected *post-hoc* tests following a two-way ANOVA were used to test for differences in the percentage of HAMD-score reduction between p11-positive and p11-negative patients.

Data were analysed employing SPSS for Windows 23.0 (SPSS Inc., Chicago, IL) or Prism 6 software from GraphPad (La Jolla, CA, USA). Results are presented as means ± SEM. All tests were used two-sided except for the application of Youden´s Index on the proof-of-concept sample in which Fisher´s exact test was one-sided; a *P*≤0.05 was considered significant.

**Supplemental figures**


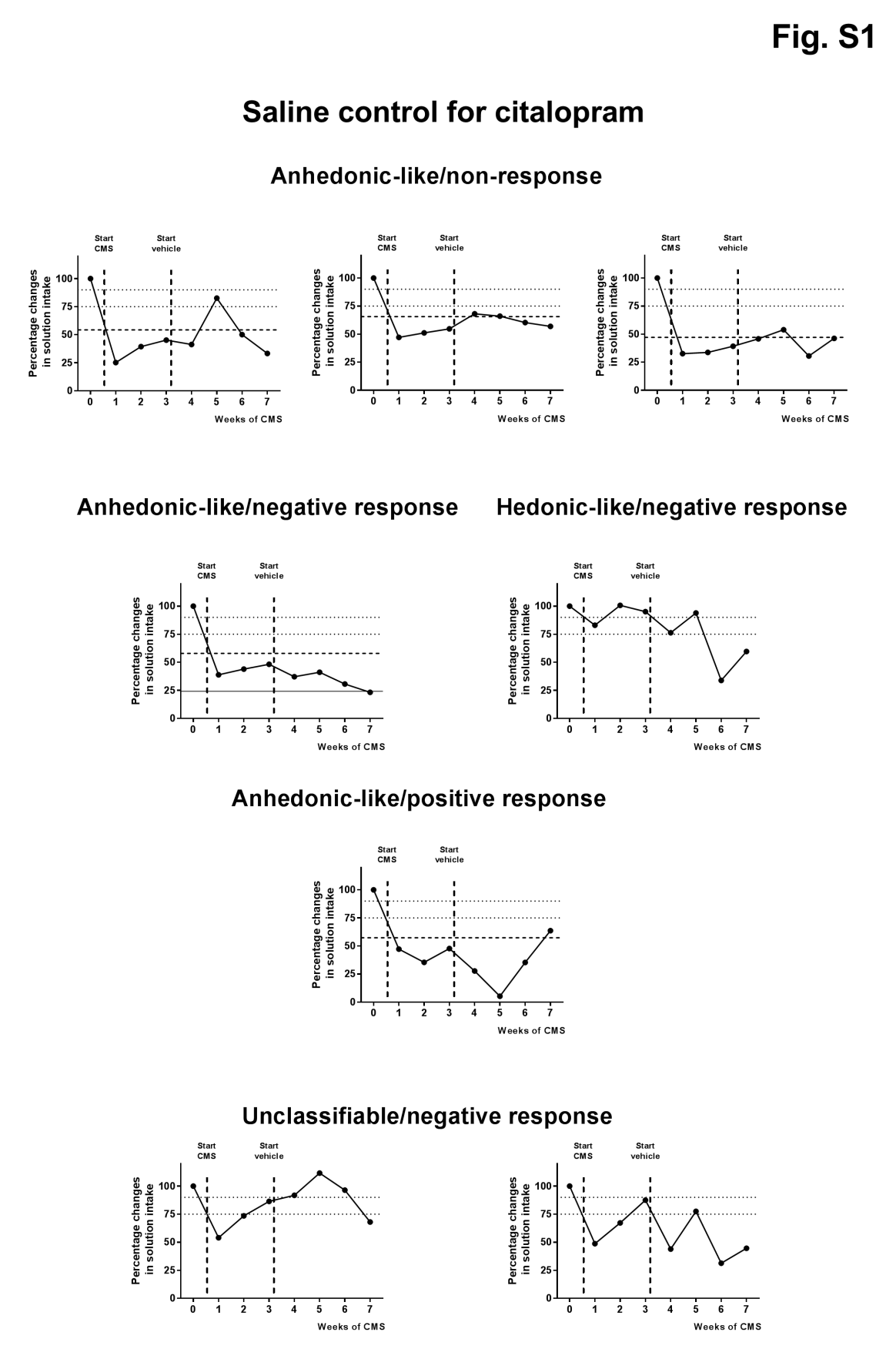


**Fig. S1.** Individual responses to chronic mild stress (CMS) and saline treatment (as control for citalopram) in the sucrose consumption test (SCT) in rats. According to their response in the SCT, rats were categorized as anhedonic-like, hedonic-like, and unclassifiable. Small dashed lines represent threshold for selection of hedonic- and anhedonic-like rats (anhedonic-like rats >25% within-subject decrease in sucrose consumption, hedonic-like rats <10% within-subject decrease in sucrose consumption). Furthermore, according to their response treatment, rats were categorized as responders, nonresponders, or negative responders (Christensen et al., 2011). Positive treatment responders were anhedonic-like animals showing a >20% within-subject increase in sucrose consumption, whereas nonresponders were rats showing a <20% within-subject increase in sucrose consumption. Moreover, treatment-induced decrease in sucrose consumption of >50% of previous anhedonic-, hedonic-like, or unclassifiable rats was characterized as negative response. Wide dashed lines represent the threshold for positive response and solid lines represent threshold for negative treatment response.


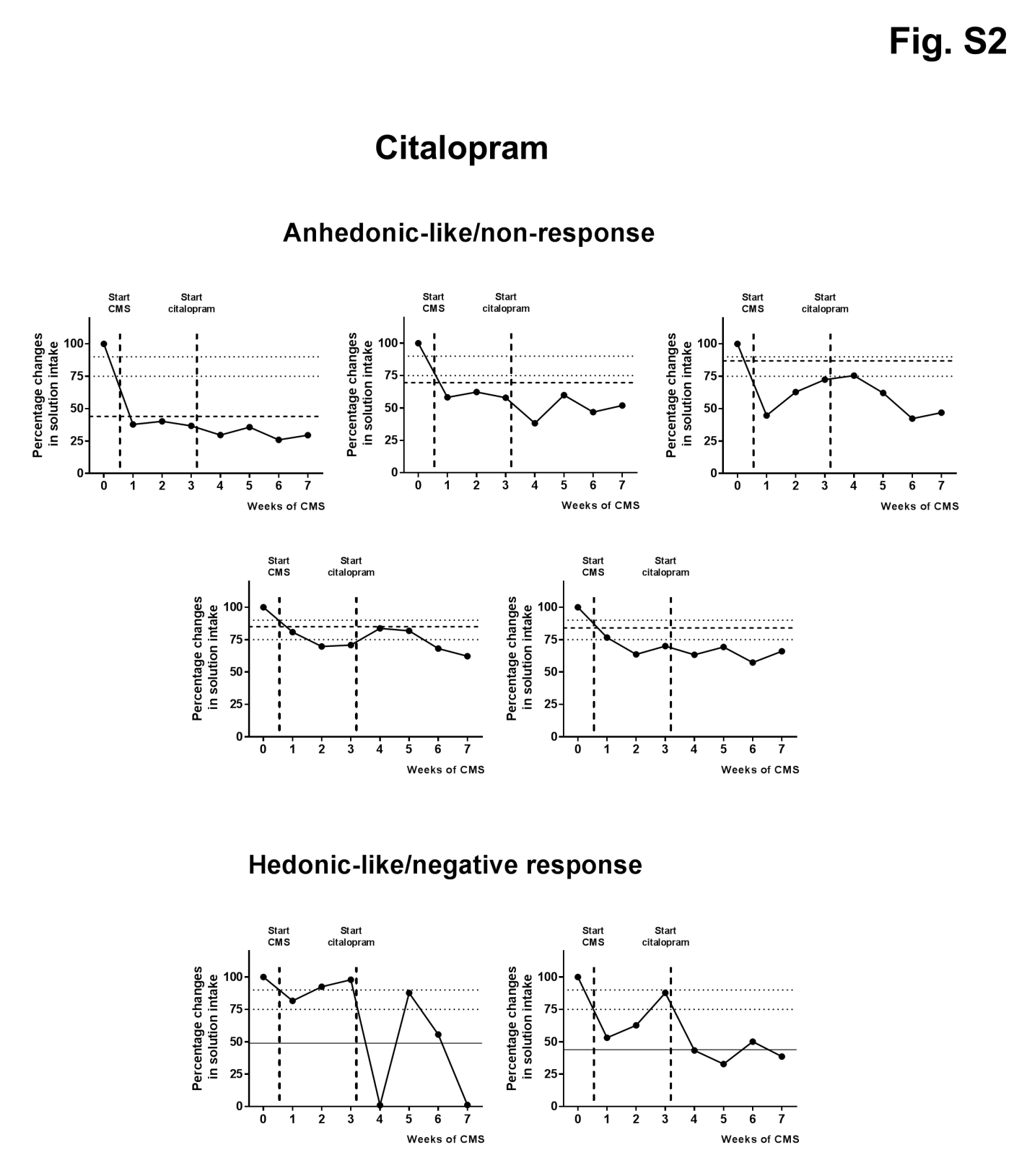


**Fig. S2.** Individual responses to chronic mild stress (CMS) and citalopram treatment in the sucrose consumption test in rats. For details see legend to Fig. S1.


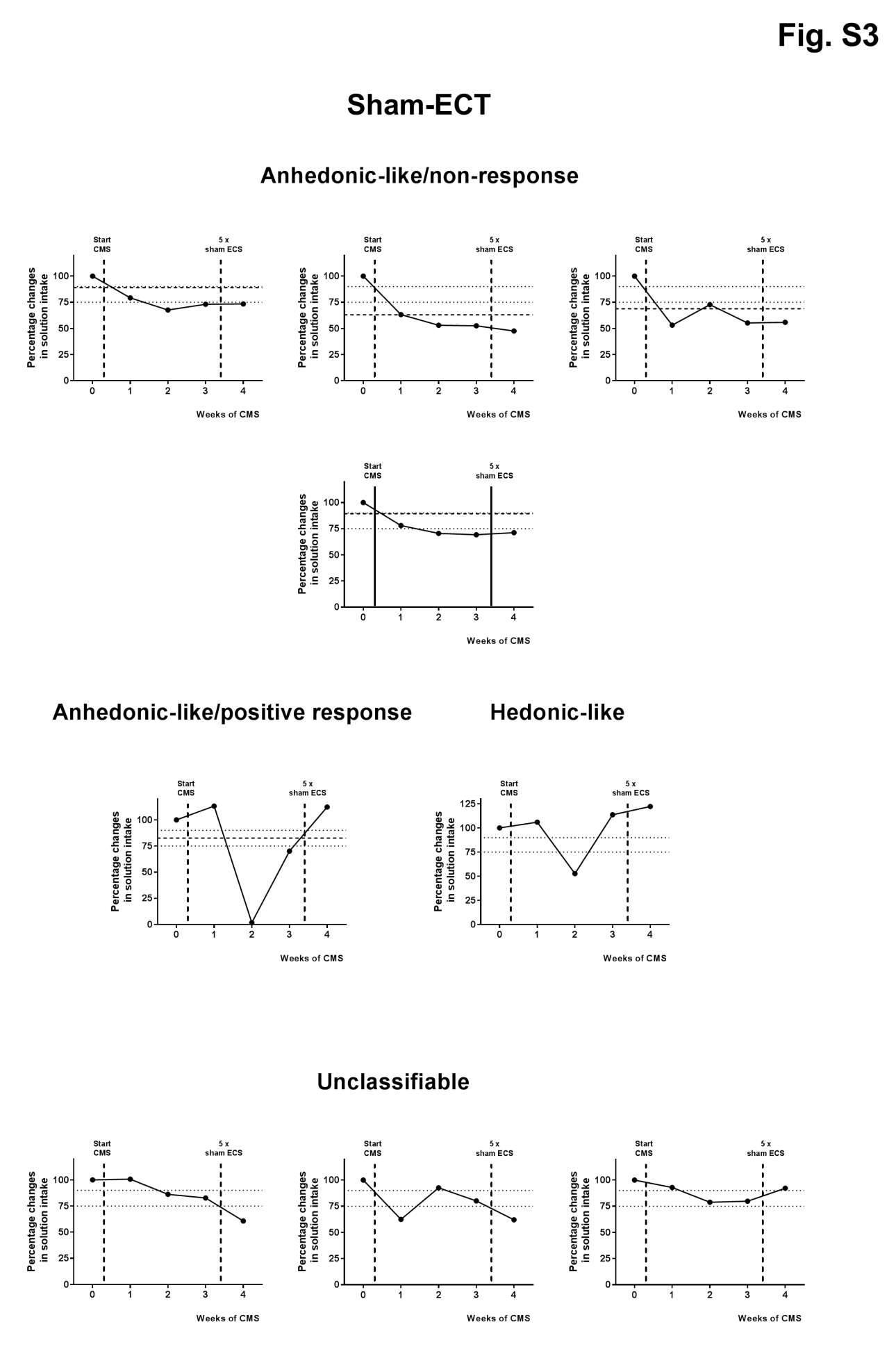


**Fig. S3.** Individual responses to chronic mild stress (CMS) and sham-ECS treatment in the sucrose consumption test in rats. For details see legend to Fig. S1.


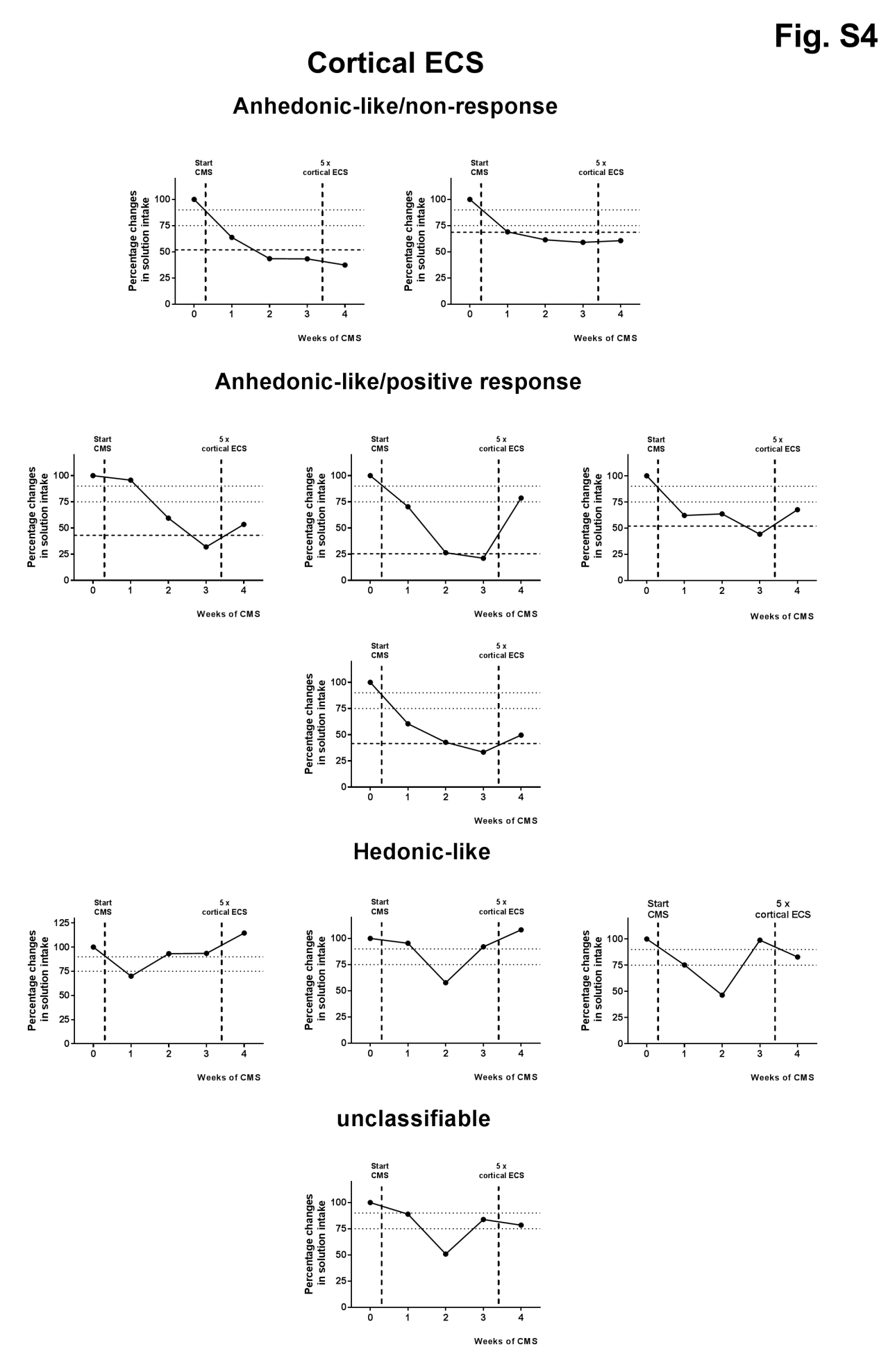


**Fig. S4.** Individual responses to chronic mild stress (CMS) and cortical ECS treatment in the sucrose consumption test in rats. For details see legend to Fig. S1.


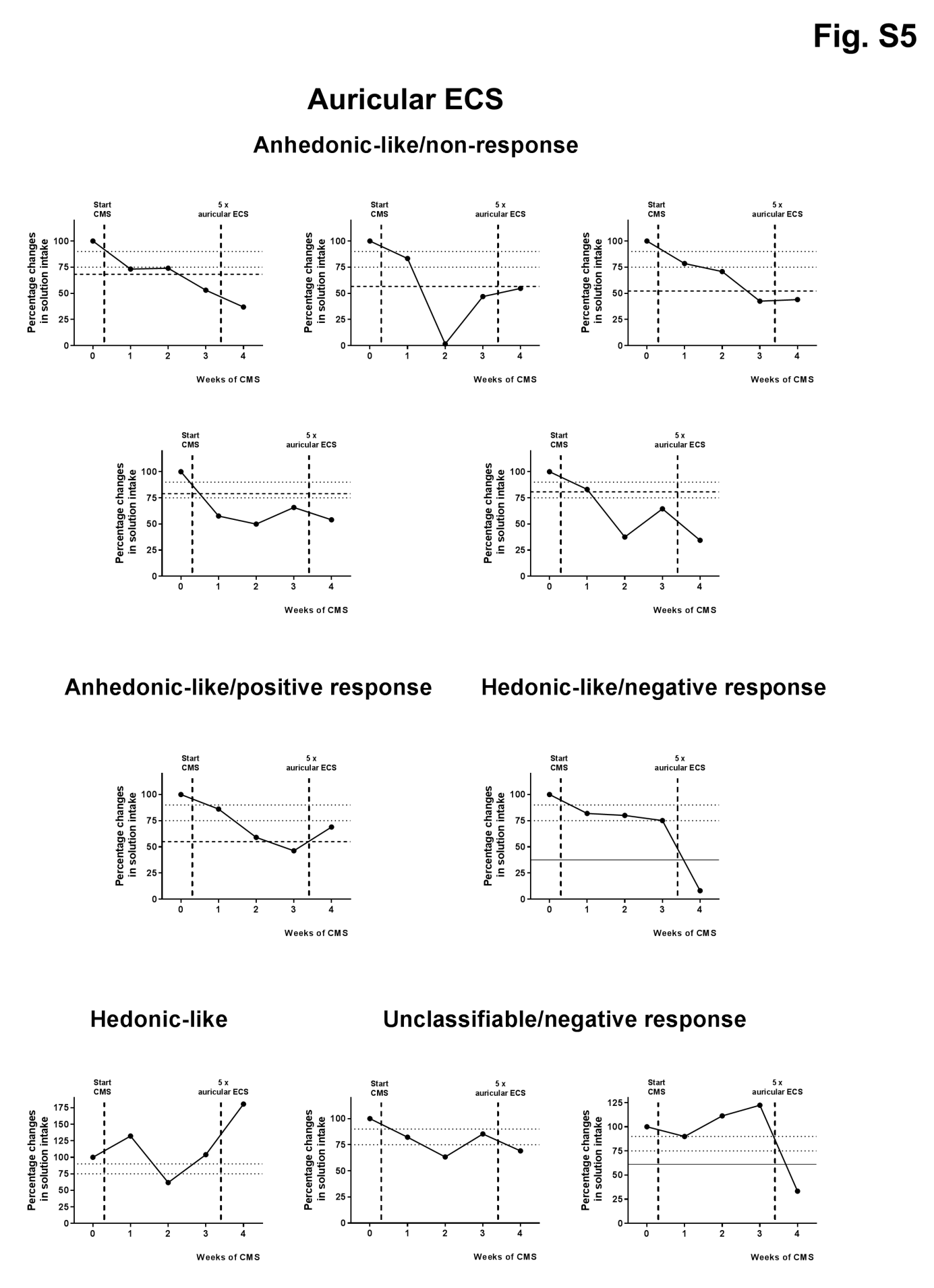


**Fig. S5.** Individual responses to chronic mild stress (CMS) and auricular ECS treatment in the sucrose consumption test in rats. For details see legend to Fig. S1.

1 *GCCTAGATGA TGAAAGGTTT GGC*TTCTGTG TGCGTGAGTT CGCTAGGACT GGAAGTGGAA

61 ACGTCTACAA AG*CATGCAAT* *GCCCTGGAA*C GGAATTCTTC TAATAAAAGA TGTATCATTT

121 TAAATG**CGCG** GAATTCTGAT TCTGGTAATT **CG**TGCACTAG AGTGTCTATT T**CG**AGGCAGA

181 GGAGGTATCA TATGACAGCT CA**CG**TCAAGG CAG**CG**TGGAG CCCTCT**CG**TG GACTCCCACC

241 CACTTTCCCA TTCAC**CG**AGG AGAGGACTGC T**CGCG**CTGCC GCTCCCCCCA CCCACCCC**CG**

301 G**CG**AGCTAGC ATGAAATCTC CCAGTCTCTG CCTAGATCAA ATGGAGCTTC TCACTGAAGG 361 **CG**TG**CG**AGTA TTACCTC**CG**C CATGCAATTT CCACTATCAA TA*ATTTAACT TCTTTGCTGA*

421 *AGAACAGGAG TA*

**Fig. S6.** Rat BDNF IV sequence. The primers are underlined and in italic, studied CpGs are underlined and bold. The arrow shows the transcription start site.

1 *TGGTAAGGCT AGGCAGGAGC TCA*GGGGCCT TAGCTTCT*GG ACTCAACACA GGAGACCCCA*

61 *GGATC*AGATC CTAGAGCAAA CTAGGTGGCC AAGCTGAGAC CTGTTCAG**CG** **CG**TTCCTTCT

121 GCTCATCCAA GAAATAATTC CTAACCCTGT AGGTTCC**CG**C AGAGG**CG**CTT GGCTCTGGTT

181 CTGGGCCAGT GTACCC**CG**CA GGAC**CG**CTGG ATTCTTATTT CTACACTCAC CCACTTTCTA

241 AAACCAAACC **CG**AGAGGAAG GG**CG**AGG**CG**C TGTATGAGAA TGCCCTGAAA TATC**CG**AGAC

301 TAGAGCATTC CTC**CG**TAGGG ACGTTTACAA TAGGGCTGTC CCTGGAGACC GGCAAAGTTA

361 *CTATGTCAGC AGAGTGAGTG GGAA*GGGTGT GTGTGTGTGT GTGTGTGTGT GTGTGTGTGT

421 GTGTGTGTGT GTGTGTGTGT GTTTTGGAGA AGAAGGGAGA G[ gap 240bp]

481 GGGAGGGCC*G CTGCCTGCCC TCCAGGCTCC TCCCA*CTC**CG** GAG**CG**CCTC**C G**CCCT**CG**GTA

541 CC**CG**CCC**CGC G**TACAAAGA**C GCGCG**ATCTT **CG**G**CG**CCAGC CCCAT**CG**CTG TGTGCCCAGC

601 TCTTCCAAAG ACTGCAG**CG**C CTCAGGGCCC AGGTGAGTCC **CG**CACTTAAT ATCTGCCCTC

661 ACCCCACAGT **CG**CTTGCTTC AGCCTCCAGC GGGGTTT**CG**C TAGTGCCT**CG** CCCCGCGGG*C*

721 *CTGAGTGAGG CCCCCTTCCA GCT*CGCAAAC TGCTTCGGAC GCCCCCAGAC CCGCTAGGCT

781 GG*AGGAAGCC AACTCTTGTC TGCAGC*

**Fig. S7.** Rat P11 sequence. The primers are underlined and in italic, studied CpGs are underlined and bold. The arrow shows the transcription start site.

1 *TTGAGACAGA GTCTCACTCT GTCACCCAGG CT*GGAGTGCA GTGGTG**CG**AT CT**CG**GCTCAC

61 TGCAACCTCT GTCTCCCAGG TTCAAG**CG**AT TCTCATGCCT CAGTCTCCTG GGATTACAGG

121 **CGCG**CATCAC CACTCC**CG**GC TAATTTTTGT ATCAGTAGAG ACAAGGTTTC ACCATGTTGG

181 CTAAGCTGGT GTTGAACTCC TGACCTGAGG TGATC**CG**CC**C G**CCT**CG**GCCT CCCAAAGTGT

241 TGGGATTACA GG**CG**TGAGCC AC**CGCG**CC**CG** GCCAGTTTTT AACACTATTA GCCACACTGA

301 AACTGAACTA TTGATCAAGT GACGCCACAC AAAGGGGTAA *ATCCCCTGTT CAACAAAGGG*

361 *TTT*GTGACGC CCCTGGGTGC TGACAAGCCA AACCGCACCC TCCCTGCGGC ACCTCGCGGG

421 CCGGTGGGGC GGGAAGCCCG GCTTCTGGGG AGGTGCCGCC CCTCCACTGG CGCAGGCCGC

481 CGAGACCCCC AGACGGACCT CCTAGGGCTA ATCTGATAGT GCCTCTGAGG TCGATAGGAC

541 TCCACGTGCC ACTCCCTGCA GGGTCATCCA GCAAGTAATT CCTAGACCCG TAGGTGGCCG

601 CAGAGCCGGT TACCT

**Fig. S8.** Human P11 sequence. The primers are underlined and in italic (F2 primer encircled), studied CpGs are underlined and bold. The arrow shows the transcription start site.


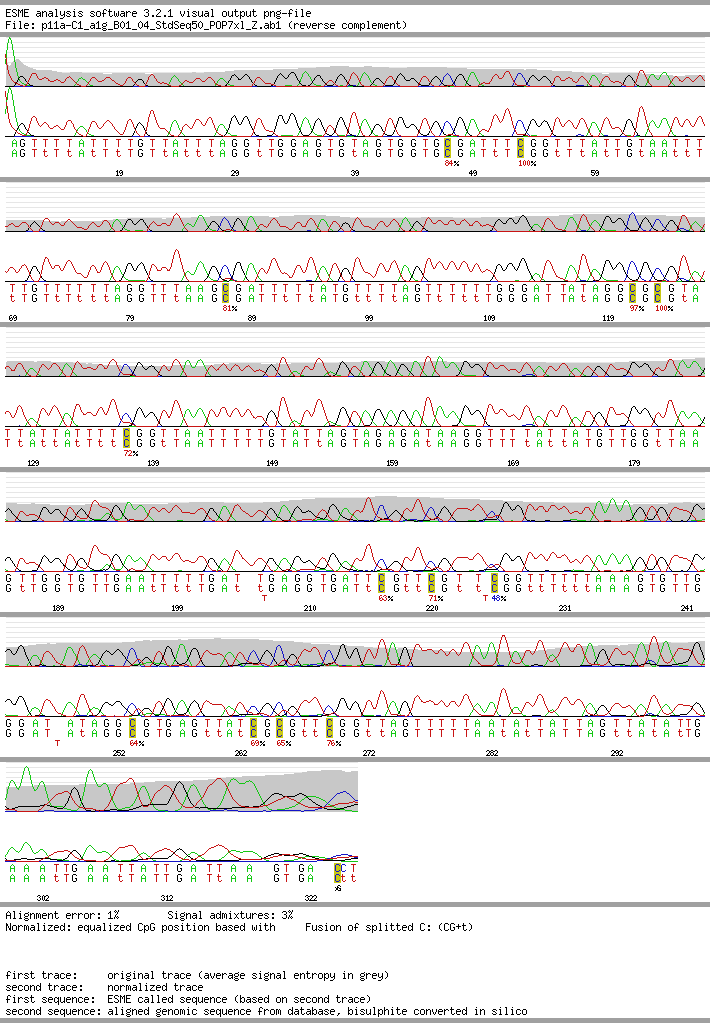


**Fig. S.9** Exemplary ESME analysis of the P11 human promoter sequence.

**Fig. S10.** The figure shows the methylation at the different CpG sites within the studied P11 promoter for the proof-of-concept study (A) and the replication sample (B). CpG´s are numbered according to their position from the beginning of the Forward1 Primer (see Figure S8).

**Supplemental tables**

**Table S1.** Patients’ characteristics of the proof-of-concept study at baseline

| Characteristics, mean (SD) | Responder (n=4) | Non-Responder (n=7) | T(df)/χ²(df) | Sig. |
| --- | --- | --- | --- | --- |
| Age, years | 45.5 (19.3) | 51.1 (16.0) | .53 (9) | .612 |
| Women, n (%) | 2 (50%) | 3 (42.9%) | .05 (1) | .819 |
| MADRS | 32.0 (12.6) | 33.7 (5.8) | .26 (3.7) ^a.^ | .810 |
| BDI | 28.3 (13.7) | 37.9 (7.9) | 1.13 (2.6) ^a.^ | .352 |
| Duration of current depressive episode, weeks | 22.7 (2.3) | 28.9 (33.2) | .49 (6.1) ^a.^ | .641 |
| Age at initial diagnosis, years | 25.5 (14.8) | 40.3 (11.4) | 1.87 (1) | .095 |
| Number of previous depressive episodes | 3.7 (2.3) | 5.0 (3.5) | .56 (9) | .609 |
| Psychotic symptoms, n (%) | 3 (75%) | 5 (71,4%) | .02 (1) | .898 |
| History of suicide attempt, n (%) | 0 (0%) | 2 (28.6%) | 1.40 (1) | .237 |
| Antidepressants, n (%) | 4 (100%) | 6 (85.7%) | .63 (1) | .428 |
| Atypical antipsychotics, n (%) | 4 (100%) | 5 (71,4%) | 1.40 (1) | .237 |

^a.^ T-value and df corrected for inhomogeneous variances

**Table S2.** Patients’ characteristics of the independent replication sample at baseline

| Characteristics, mean (SD) | Responder (n=50) | Non-Responder (n=15) | T(df)/χ²(df) | Sig. |
| --- | --- | --- | --- | --- |
| Age, years | 50.2 (17.3) | 51.7 (16.7) | .30 (63) | .765 |
| Women, n (%) | 19 (38%) | 8 (53.3%) | 1.12 (2) | .291 |
| HAMD | 28.5 (1.9) | 28.5 (1.3) | .61 (63) | .951 |
| Duration of current depressive episode, weeks | 17.2 (11.1) | 25.6 (12.7) | 2.50 (63) | .015* |
| Age at initial diagnosis, years | 31.6 (14.0) | 29.6 (15.9) | .46 (63) | .644 |
| Number of previous depressive episodes | 5.5 (4.2) | 7.5 (5.0) | 1.53 (63) | .132 |
| Psychotic symptoms, n (%) | 6 (12%) | 2 (13.3%) | .02 (1) | .890 |
| Antidepressants, n (%) | 45 (91.8%) | 12 (85.7%) | .47 (1) | .491 |
| Atypical antipsychotics, n (%) | 21 (42,9%) | 7 (50%) | .23 (1) | .635 |
| Leucocyte count [thsd./µl] | 7.8 (1.9) | 9.1 (2.7) | 1.61(17.2) ^a.^ | .126 |

*P<0.05 Student’s t-test; ^a.^ T-value and df corrected for inhomogeneous variances

**Table S3.** Allocation of rats to treatment groups. In both experiments, the animals were randomly divided into the treatment groups (based on pretreatment SCT data) and it was ensured that the numbers of anhedonic-like, hedonic-like and unclassifiable rats were almost the same in all treatment groups.

| Treatment group | No. of anhedonic-like animals after CMS | No. of hedonic-like animals after CMS | No. of unclassifiable animals after CMS |
| --- | --- | --- | --- |
| **Experiment I** |  |  |  |
| Vehicle | 5 | 1 | 2 |
| Citalopram | 5 | 2 | 0 |
| **Experiment II** |  |  |  |
| Sham | 5 | 1 | 3 |
| Cortical ECS | 6 | 2 | 1 |
| Auricular ECS | 6 | 2 | 2 |

**Table S4:** Overview of all analyzed depression-associated symptoms with the respective behavioral tests in the CMS model in rats. Abbreviations: ECS, electroconvulsive stimulation; FST, forced swim test; NIH, novelty-induced hypophagia; OFT, open field test; SCT, sucrose consumption test; SIT, social interaction test.

**___________________________________________________________________________**

**Symptom group Symptom item**

**_________________________________________________________________________________**

I Anhedonic-like behavior: (1) Percentage change in sucrose consumption (SCT)

II General well-being: (2) Bodyweight changes

III Anxiety-related behavior: (3) Latency to eat food in a novel surrounding (NIH) (4) Amount of eaten food in a novel surrounding (NIH) (5) Time spent in the center of an open arena (OFT) (6) Grooming activity (OFT)

IV Locomotor activity: (7) Distance moved in an open arena (OFT)

V Social interaction: (8) Mean body distance between unfamiliar rats (SIT) (9) Time two unfamiliar rats spent in body contact (SIT)

Additionally assessed in rats with ECS

VI Behavioral despair: (10) Duration of immobility in escape-less situation (FST)

**_________________________________________________________________________________**

**Table S5.** Primer sets rat p11 quantitative RT-PCR*

| RT-PCR | 619-p11rat_RT_F:  620-p11rat_RT_R: | CAGGTTTCAACAGATTC  GTCCAGGTCTTTCATTA |
| --- | --- | --- |

* Rattus norvegicus S100 calcium binding protein A10 (S100a10), mRNA

NCBI Reference Sequence: NM_031114.1

**Table S6.** Primer sets rat p11 Promoter on Chromosome 2 Fragment A:193891825-193892120*

| PCR I | 550-p11rat_prom_F1:  552-p11rat_prom_R1: | TGGTAAGGTTAGGTAGGAGTTTA  TTCCCACTCACTCTACTAACATAA |
| --- | --- | --- |
| PCR II | 551-p11rat_prom_F2:  552-p11rat_prom_R1: | GGATTTAATATAGGAGATTTTAGGATT  TTCCCACTCACTCTACTAACATAA |

* According to Rnor 6.0 (Rat Genome Sequencing Consortium Date: 2014/07/01)

GenBank Assembly ID: GCA_000001895.4 (latest)

Primer sets rat p11 Exon I Chromosome 2 Fragment B:193892497-193892700*

| PCR I | 554-p11rat_F1:  556-p11rat_R1: | GTTGTTTGTTTTTTAGGTTTTTTTTA  ACTACAAACAAAAATTAACTTCCT |
| --- | --- | --- |
| PCR II | 554-p11rat_F1:  557-p11rat_R2: | GTTGTTTGTTTTTTAGGTTTTTTTTA  AACTAAAAAAAAACCTCACTCAAA |

* According to Rnor 6.0 (Rat Genome Sequencing Consortium Date: 2014/07/01)

GenBank Assembly ID: GCA_000001895.4 (latest)

**Table S7.**

Primer sets rat BDNF Exon IV on Chromosome 3:100786624 to 100787056 *

| PCR I | 391-BDNFr-E4-F3:  394-BDNFr-E4-RC1: | GTTTAGATGATGAAAGGTTTGGT  TACTCCTATTCTTCAACAAAAAAATTAAAT |
| --- | --- | --- |
| PCR II | 392-BDNFr-E4-F2:  394-BDNFr-E4-RC1: | ATAAAGTATGTAATGTTTTGGAA  TACTCCTATTCTTCAACAAAAAAATTAAAT |

# * According to Rnor_6.0 Primary Assembly (Rat Genome Sequencing Consortium; Date: 2014/07/01) GenBank assembly accession: GCA_000001895.4 (latest)

**Table S8.** Primer sets human p11 Promoter on Chromosome 1: 151994448 to 151994810*

| PCR I  Fragment A | 510-P11A_F1:  512-P11A_R1: | TTGAGATAGAGTTTTATTTTG  AAACCCTTTATTAAACAAAAAAT |
| --- | --- | --- |
| PCR II  Fragment A | 511-P11A_F2:  512-P11A_R1: | AGTTTTATTTTGTTATTTAGGTT  AAACCCTTTATTAAACAAAAAAT |

* According to GRCh38.p2 Primary Assembly (Genome Reference Consortium Human Build 38 patch release 2; Date: 2014/12/05) GenBank assembly accession: GCF_000001405.33 (latest)

**Supplemental references**

1. Theilmann,W, Löscher,W, Socala,K, Frieling,H, Bleich,S, Brandt,C (2014): A new method to model electroconvulsive therapy in rats with increased construct validity and enhanced translational value.  *J Psychiatr.Res* 53: 94-98.

2. Theilmann,W, Kleimann,A, Rhein,M, Bleich,S, Frieling,H, Löscher,W et al (2016): Behavioral differences of male Wistar rats from different vendors in vulnerability and resilience to chronic mild stress are reflected in epigenetic regulation and expression of *p11* . *Brain Res.* 1642: 505-515..

3. Langer,M, Brandt,C, Löscher,W (2011): Marked strain and substrain differences in induction of status epilepticus and subsequent development of neurodegeneration, epilepsy, and behavioral alterations in rats. *Epilepsy Res* 96: 207-224.

4. Willner,P, Towell,A, Sampson,D, Sophokleous,S, Muscat,R (1987): Reduction of sucrose preference by chronic unpredictable mild stress, and its restoration by a tricyclic antidepressant. *Psychopharmacology (Berl)* 93: 358-364.

5. Jayatissa,MN, Bisgaard,C, Tingstrom,A, Papp,M, Wiborg,O (2006): Hippocampal cytogenesis correlates to escitalopram-mediated recovery in a chronic mild stress rat model of depression. *Neuropsychopharmacology* 31: 2395-2404.

6. Christensen,T, Bisgaard,CF, Wiborg,O (2011): Biomarkers of anhedonic-like behavior, antidepressant drug refraction, and stress resilience in a rat model of depression. *Neuroscience* 196: 66-79.

7. Porsolt,RD, Le Pichon,M, Jalfre,M (1977): Depression: a new animal model sensitive to antidepressant treatments. *Nature* 266: 730-732.

8. Hall,C, Ballachey,EL (1932): A study of the rat's behavior in a field. A contribution to method in comparative psychology. *University of California Publications in Psychology* 6: 1-12.

9. Bodnoff,SR, Suranyi-Cadotte,B, Aitken,DH, Quirion,R, Meaney,MJ (1988): The effects of chronic antidepressant treatment in an animal model of anxiety. *Psychopharmacology (Berl)* 95: 298-302.

10. Shephard,RA, Broadhurst,PL (1982): Effects of diazepam and picrotoxin on hyponeophagia in rats. *Neuropharmacology* 21: 771-773.

11. Santarelli,L, Saxe,M, Gross,C, Surget,A, Battaglia,F, Dulawa,S et al (2003): Requirement of hippocampal neurogenesis for the behavioral effects of antidepressants. *Science* 301: 805-809.

12. File,SE, Hyde,JR (1978): Can social interaction be used to measure anxiety? *Br.J.Pharmacol.* 62: 19-24.

13. Kusmider,M, Solich,J, Palach,P, Dziedzicka-Wasylewska,M (2007): Effect of citalopram in the modified forced swim test in rats. *Pharmacol.Rep.* 59: 785-788.

14. Koenigs,M, Grafman,J (2009): The functional neuroanatomy of depression: distinct roles for ventromedial and dorsolateral prefrontal cortex. *Behav.Brain Res.* 201: 239-243.

15. Melas,PA, Rogdaki,M, Lennartsson,A, Bjork,K, Qi,H, Witasp,A et al (2012): Antidepressant treatment is associated with epigenetic alterations in the promoter of P11 in a genetic model of depression. *Int.J.Neuropsychopharmacol.* 15: 669-679.

16. Schmidt,EF, Warner-Schmidt,JL, Otopalik,BG, Pickett,SB, Greengard,P, Heintz,N (2012): Identification of the cortical neurons that mediate antidepressant responses. *Cell* 149: 1152-1163.

17. Montgomery,SA, Asberg,M (1979): A new depression scale designed to be sensitive to change. *Br.J.Psychiatry* 134: 382-389.

18. Hamilton,M (1960): A rating scale for depression. *J.Neurol.Neurosurg.Psychiatry* 23: 56-62.

19. Rhein,M, Muschler,MR, Krauss,JK, Bleich,S, Frieling,H, Schwabe,K (2013): Hypomethylation of neuregulin in rats selectively bred for reduced sensorimotor gating. *SCHIZOPHR.RES.* 150: 262-265.

20. Kleimann,A, Kotsiari,A, Sperling,W, Groschl,M, Heberlein,A, Kahl,KG et al (2015): BDNF serum levels and promoter methylation of BDNF exon I, IV and VI in depressed patients receiving electroconvulsive therapy. *J.Neural Transm.(Vienna.)* 122: 925-928.

21. Hellemans,J, Mortier,G, De Paepe,A, Speleman,F, Vandesompele,J (2007): qBase relative quantification framework and software for management and automated analysis of real-time quantitative PCR data. *Genome Biol.* 8: R19.

22. Lewin,J, Schmitt,AO, Adorjan,P, Hildmann,T, Piepenbrock,C (2004): Quantitative DNA methylation analysis based on four-dye trace data from direct sequencing of PCR amplificates. *Bioinformatics.* 20: 3005-3012.

23. Rantamaki,T, Hendolin,P, Kankaanpaa,A, Mijatovic,J, Piepponen,P, Domenici,E et al (2007): Pharmacologically diverse antidepressants rapidly activate brain-derived neurotrophic factor receptor TrkB and induce phospholipase-Cgamma signaling pathways in mouse brain. *Neuropsychopharmacology* 32: 2152-2162.

24. Hillemacher,T, Weinland,C, Lenz,B, Kraus,T, Heberlein,A, Glahn,A et al (2015): DNA methylation of the LEP gene is associated with craving during alcohol withdrawal. *Psychoneuroendocrinology* 51: 371-377.
